# The Effect of Abnormal Reproductive Tract Discharge on the Calving to Conception Interval of Dairy Cows

**DOI:** 10.3389/fvets.2019.00374

**Published:** 2019-10-22

**Authors:** Madeleine J. Hay, Allan J. Gunn, Angel Abuelo, Victoria J. Brookes

**Affiliations:** ^1^Graham Centre for Agricultural Innovation (NSW Department of Primary Industries and Charles Sturt University), Wagga Wagga, NSW, Australia; ^2^School of Animal and Veterinary Sciences, Faculty of Science, Charles Sturt University, Wagga Wagga, NSW, Australia; ^3^Department of Large Animal Clinical Sciences, College of Veterinary Medicine, Michigan State University, East Lansing, MI, United States

**Keywords:** dairy, cow, calving to conception, endometritis, Holstein, Friesian, survival analysis

## Abstract

Prolonged calving-to-conception interval (CCI) can increase economic loss in cattle. We investigated the effect of post-calving abnormal reproductive tract discharge (ARTD) on CCI in dairy cows and quantified the relationship of ARTD and associated risk factors with CCI. The source population was dairy cows that calved in the study period on three pasture-based, year-round calving farms in the Riverina, NSW, Australia. Farm records and records from veterinarians' visits were analyzed. ARTD was defined as the presence of reproductive tract discharge according to the following classification: per vaginum purulent discharge ≥21 days post-calving or mucopurulent discharge >26 days post-calving. The incidence of ARTD was calculated. A Kaplan-Meier survivor function was used to estimate median time to conception post-calving dependent on the presence or absence of ARTD. Mixed effects Cox-proportional hazard models were used to estimate the direct and indirect effects of ARTD, and other potential risk factors on CCI such as body condition score (BCS), ambient temperature, and milk yield. Model structures were guided by a directed acyclic graph of potential risk factors for ARTD. The incidence of ARTD in lactations was 16% (95% CI 13.8–18.5%) and did not differ significantly between the three farms (*P* > 0.05). The median CCI was 176 and 118 days for lactations with and without ARTD, respectively (*P* < 0.01). The rate of pregnancy following calving in cows with ARTD was significantly decreased relative to the rate of pregnancy in cows without (total effect hazard ratio = 0.62, se = 0.18, *P* = 0.01). High peak milk yield (>32 L) and parity >2 also significantly extended CCI. We did not observe an effect of BCS or ambient temperature on CCI. The incidence of ARTD in the current study was consistent with clinical endometritis (considered a major source of ARTD) reported in other studies. In contrast—and despite regular veterinary assessment and treatment of on the farms in this study—ARTD extended CCI. Evaluation of the economic impact of ARTD on dairy farms in this region is warranted, and methods to identify high risk cows and develop effective interventions are required.

## Introduction

Minimizing the duration of the calving-to-conception interval (CCI)—the period between parturition and the following conception—of dairy cows has been found to be economically beneficial due to a variety of factors. These include increased milk yield relative to labor and feed costs (1–7), an increased number of calves (3, 5) and lifetime productive days (8), as well as reduced breeding costs (3, 8–11) and culling rates (3, 4, 8, 9). Post-partum uterine health is a critical factor when considering the fertility of dairy cows and the consequent duration of the CCI (12–14). Many studies have demonstrated the negative impact of endometritis on the reproductive performance of dairy cows and subsequent increased CCI (15–17).

Endometritis is defined as inflammation of the endometrium (18) and can be categorized as clinical or subclinical (19). Madoz et al. (20) define subclinical endometritis as having ≥8% neutrophils in uterine cytology samples (collected using a cytobrush) between days 21 and 33 postpartum, having ≥6% neutrophils between 34 and 47 days or ≥4% after 47 days postpartum. However, there is a lack of agreement in the literature about the normal neutrophil percentage at increasing days postpartum (21). Clinical endometritis is indicated by the presence of abnormal uterine discharge and it is considered that the most common cause of abnormal vaginal or vulval discharge in the post-calving period is clinical endometritis (12). Therefore, on farm diagnosis of clinical endometritis relies on detection of abnormal reproductive tract discharge (ARTD) which is defined as purulent (>50% pus) discharge in the vagina ≥21 days postpartum, or mucopurulent (some purulent material, but overall ≥50% mucus) discharge in the vagina when >26 days postpartum (12, 15, 19, 22). Cases of abnormal reproductive tract discharge that occur <21 days postpartum are not classified as clinical endometritis because the varying appearances of normal lochia would interfere with the diagnosis, resulting in many false positives by including cows that are spontaneously recovering from normal postpartum bacterial contamination (19).

The detrimental effect of endometritis on reproductive performance—measured as increased CCI—occurs due to direct adverse effects on the uterine environment as well as disruption of the hormonal pathways involved in the hypothalamic (GnRH)-pituitary (FSH and LH)—reproductive tract (progesterone, estrogen, inhibin, and prostaglandin) axis and subsequent effects on ovulation, conception and embryo survival (12, 23–25). These effects are mediated by bacterial products such as lipopolysaccharides, by inflammatory mediators such as nitric oxide and cytokines, and by oxidative stress, which can affect the functionality of the hypothalamus, pituitary, ovary, uterus, and spermatozoa (12, 23).

In addition, there are also risk factors other than endometritis for prolonged CCI, such as retained fetal membranes and metabolic disease. These risk factors can also contribute to the occurrence of endometritis. For example, retained fetal membranes are a risk factor for endometritis (26–33), and an association has been found between hypocalcaemia and a higher incidence and severity of endometritis (34, 35). Hypocalcaemia contributes to periparturient immune cell dysfunction (36, 37), which can limit the ability of the cow to resolve uterine contamination. Also, hypocalcaemia can cause reduced uterine contractions during parturition and subsequently delayed uterine involution postpartum, which are both risk factors for endometritis (12, 38, 39). A study by Bacha and Regassa (40) also found an association between mastitis and subclinical endometritis.

Body condition score (BCS), energy deficit, and high milk yield influence CCI and the occurrence of endometritis (38, 40–47). There is a positive correlation between high milk yields in the current or previous lactation and a prolonged interval to first service (46). Energy deficit in the postpartum period can lead to a dysregulated immune response (48) and delayed uterine involution, which can increase the likelihood of endometritis (15, 35, 49). In addition to the association between low BCS and poor reproductive performance, Le Blanc (50) found that a high BCS might also be associated with endometritis.

The association between parity and endometritis in dairy cows is not clear. Whilst some studies found no association between parity and endometritis (43, 51), others found a higher prevalence of endometritis in primiparous groups (27, 29, 39, 44, 52). However, Kim and Kang (26) found endometritis to be less common in primiparous cows due to faster uterine involution compared with multiparous cows. In some reports higher parity has been found to be a risk factor of clinical endometritis (30, 53). Hence, despite some mixed results in the literature, it appears that parity is associated with the risk of purulent vaginal discharge with older cows exhibiting less uterine elasticity and slower uterine involution, which may lead to more persistent infections (15).

Environmental and management factors such as season, oestrus detection and artificial insemination also influence CCI. Hotter seasons resulted in reduced oestrus expression and consequently, reduced conception rates in some studies (45, 54) and Bruun et al. (29) and Ghavi Hossein-Zadeh et al. (39) both found there was an increase in metritis (a risk factor for endometritis) when cows were calving in the winter months. Consistent with these findings, primiparous cows reportedly had higher levels of metritis in cooler seasons; however, in contrast, season had no effect on multiparous cows (33). Others found no association between calving season and endometritis (26, 43). Lastly, conception rates of dairy cows vary between artificial insemination technicians (55) and inadequate ability to detect oestrus has a strong association with an increased CCI (56).

Herd health management programs (HHMP) have become commonplace in many livestock systems worldwide, including dairy, as the economic margin between farm income and production costs has decreased (57). The aim is to support farmers in improving herd performance by moving veterinary involvement toward preventive health at the herd level (58). Given the economic benefits of reducing CCI on dairy farms, regular herd fertility assessments can be included in HHMP (“routine visits”) by veterinary practitioners. During these visits, individual cows are examined to assess uterine involution and health, and return to reproductive cyclicity following calving, with the aim of efficient re-breeding to reduce CCI. Early detection and resolution of post-calving problems, such as ARTD, is part of this examination. Perceived benefits include improved herd level fertility and consequentially, increased financial gain (59).

Overall, there are many factors that influence CCI, either directly or indirectly, and determining the relative importance of these factors is difficult. The objective of the following study was to investigate the incidence of ARTD in dairy cows on farms on which regular veterinary examinations post-calving occurred (in the Riverina, New South Wales, Australia), and to explore the relationship of ARTD and other risk factors with CCI. To attempt to untangle factors that influence CCI relative to the detection and treatment of ARTD, we use directed acyclic graphs (DAGs) to inform investigation of risk factors for CCI using mixed-effects Cox proportional hazard models.

## Materials and Methods

### Study Population and Data Collection

The source population was Holstein-Friesian cows on three dairy farms in the Riverina region of New South Wales, Australia. This was a convenience sample due to selection based on farms which had regular herd health visits by authors AG and AA. Each farm implemented a pasture-based, year-round calving system. The study population was cows which calved during the study period (January 2015—August 2017 inclusive). Farms 1, 2, and 3 maintained approximately 140, 65, and 240 lactating cows annually during the study period, respectively. Artificial insemination was used for all breeding on each farm, and all farms observed a voluntary waiting period for submission for insemination of 50 days to account for uterine involution. Retrospective data were obtained from fortnightly veterinary visits to each farm. All cows were initially scheduled for examination to identify abnormal reproductive tract discharge (ARTD) at ~30–40 days in milk (DIM), then again at ~50–60 DIM. If ARTD was detected, the cow was re-examined at the next veterinary visit. The examinations were conducted by one of two cattle medicine and reproduction specialist-trained veterinarians (AG and AA) who attended all farms regularly throughout the study period.

ARTD was determined by the color and consistency of uterine discharge withdrawn on a gloved hand during *per vaginum* reproductive tract examination. To be consistent with previous authors' definitions of ARTD that was most likely associated with clinical endometritis (12, 15, 19, 22), we defined ARTD as purulent (≥50 % pus) discharge diagnosed ≥21 DIM, or mucopurulent (some purulent material, but overall, ≥50% mucus) discharge diagnosed >26 and <80 DIM. Cows diagnosed with ARTD were administered 500 μg cloprostenol by intramuscular injection if a corpus luteum (CL) was identified ultrasonographically. If no CL was present, cows were re-examined and cloprostenol administered at the next visit that a CL was detected. This treatment was repeated if the ARTD was unresolved on subsequent reproductive tract examinations during veterinary visits until ARTD was no longer detected.

Data that were recorded during veterinary visits by veterinarians included calving date, DIM, pregnancy status, days in-calf (pregnant, diagnosed between 30 and 60 days gestation), the presence and characteristics of uterine discharge, reproductive cyclicity status (the presence of a CL), body condition score (BCS), and concurrent disease (periparturient disease; PPD). BCS was measured to the nearest half point (0.5) for each cow, using a previously described 5-point system (60). BCS was not measured on Farm 3. Data that were also acquired from farm records included parity (not available for Farm 3), PPD if not noted on visit records (for example, ketosis, hypocalcaemia, displaced abomasum, and lameness), peak milk yield (the highest recorded daily yield recorded for each individual lactation, from monthly milk recording information; not available for Farm 3). We also consulted records from the Australian Bureau of Meteorology (61) to identify the mean maximum daily ambient temperature in the previous month to the last recorded insemination in each lactation. The insemination date associated with the first positive pregnancy diagnosis was used to estimate the CCI. Pregnancy losses that occurred after this first detected conception were considered to have causes other than endometritis.

### Data Management and Descriptive Statistics

Data were cleaned to remove duplications and correct inconsistencies (for example, if insemination date was not consistent with days in calf, farm managers were consulted to identify correct dates). All analyses were performed using the software R (62) and packages “plyr” (63), “survival” (64), “tidyverse” (65), “survminer” (66), and “coxme” (67).

Summary statistics described the number of lactations and the incidence risk of ARTD in individual cows and in all lactations. Summary statistics also described the distributions of parity, BCS, peak milk yield and the types and risk of PPD. Due to the potential increased submissions for insemination in cows with ARTD in the same lactation (“repeat breeders”), we also summarized the number of inseminations/cow and assessed whether there was a statistically significant difference between cows with and without ARTD.

Due to the presence of right censored data (pregnancy not observed during a lactation) and the likely changing population rate of pregnancy throughout the lactation, survival analysis methods were used to investigate the influence of ARTD and other potential risk factors on CCI. Right censoring was defined when a cow was not recorded as pregnant but no longer appeared in the dataset. A Kaplan-Meier survivor function was used to visually assess and estimate median time to pregnancy post-calving, dependent on the presence or absence of ARTD in the lactation. Boxplots of the duration to censoring were compared to determine if there was a difference in censoring between groups (which could then have been related to the variable of particular interest; presence or absence of ARTD).

### Analysis of Risk Factors

**Figure 1:**
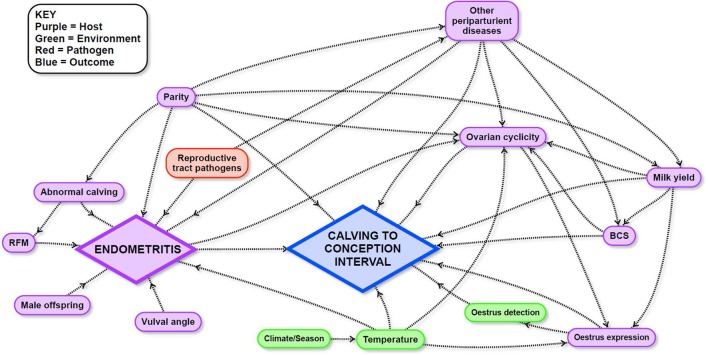
Directed acyclic graph used to inform survival analyses to identify the influence of endometritis and other potential risk factors in the peri- and post-partum period on calving to conception interval in a study of dairy cows in the Riverina, New South Wales, Australia.

Mixed effects Cox-proportional hazard models were used to estimate the effects of potential risk factors on CCI. Potential causal pathways and links between these variables and CCI were described and illustrated using a directed acyclic graph (DAG; Figure 1) in which a direct effect of a predictor variable is illustrated by a single arrow from the variable to the outcome, and an indirect effect is illustrated by one or more intervening variables on the pathway to the outcome. The combined effect of the direct and indirect pathways is the total effect of a variable. Detailed explanations about the interpretation of causal diagrams are available in Dohoo et al. (68). The DAG for the current study was informed by a narrative review of the literature (Supplementary Dataset 1) followed by extensive discussions between all authors to define the final DAG. Variables that were dependent on farm of origin—for example, nutrition—were treated as “farm effects” and were not included in the DAG because cow-level variables which could be generalized to other farms were the focus of the current study.

The exposure variable of primary interest in this study was the presence of ARTD in the post-calving period. The direct and total effects of this variable were investigated. The total effect quantifies the influence of the presence or absence of ARTD in the post-calving period on CCI, including its effects mediated via intervening variables. The total effect is therefore, the value of greatest interest. The direct effect of ARTD in the post-calving period on CCI was also investigated to determine the relative influence of the effect of ARTD via intervening variables. In addition, the total effects of other variables of interest (BCS, parity, peak milk yield, and temperature) were quantified to assess their influence on CCI relative to the total effect of ARTD. We considered that the effects of BCS, parity, and milk yield were unlikely to be linear and therefore, investigated their effects in plausible biological categories (Table 1). The cut-off for high or low milk yield was defined as the median peak milk yield of all cows in the study. Each model included the variable of interest and the minimal sufficient adjustment set of covariates for either direct or total effects (7 models listed in Table 1), which were identified using the software Dagitty (69).

**Table 1:** Exposure variables of interest and minimal sufficient adjustment sets of covariates used in mixed-effects Cox-proportional hazards models to quantify direct and indirect effects on calving to conception interval in dairy cows in the Riverina, Australia.

**Exposure variable and categories**	**Minimal sufficient adjustment set of covariates**	**Effect measured**
ARTD	Other periparturient diseases, parity, temperature	Total
ARTD	BCS, milk yield, other periparturient diseases, ovarian cyclicity, parity, temperature	Direct
BCS (≤2, 2 ≤ 4, >4)	Milk yield, other periparturient diseases	Total
Peak Milk Yield (low ≤32 L/day, high >32 L/day)	Other periparturient diseases, parity	Total
Parity (low ≤2, high >2)	—	Total
Temperature	—	Total
Peri-parturient disease	Parity, reproductive tract pathogens	Total

*ARTD, abnormal reproductive tract discharge; BCS, body condition score*.

Natural clustering within the study data (farm and repeated measurements of individual cows) was modeled as nested random effects to account for the effects of unmeasured farm-level variables such as diet and cow-level variables such as individual susceptibility. The assumption of proportional hazards and potential existence of time dependency of variables in the models of direct and total effects ARTD on CCI were investigated by assessing Schoenfeld residuals, plots of predicted and observed data, and cumulative log plots of observed data.

## Results

### Summary Statistics

Following data preparation, this study included 232, 93, and 334 cows (total 659) during the study period from Farms 1, 2, and 3, respectively. The first and last observation dates recorded during the study were 06/01/15 and 15/08/17, respectively. There were a total of 977 lactations included in the study, with 361, 141, and 475 lactations from Farms 1, 2, and 3, respectively. The number of lactations per cow during the study period ranged from 1 to 3 (median 2 lactations/cow). The mean duration of monitored periods was 125 days (95 % range 40–343 days).

**Figure 2:**
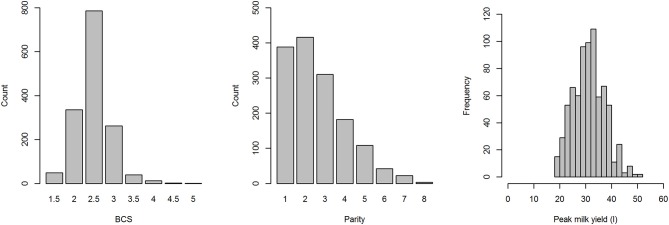
Distributions of body condition score (BCS), parity and peak mild yield of cows from farms 1 and 2 in a study to identify the influence of abnormal reproductive tract discharge and other potential risk factors on the calving to conception interval in dairy cows in the Riverina, NSW, Australia.

On farms for which the parity of each cow was available (Farms 1 and 2), parity ranged from 1 to 8 (median = 2; Figure 2). BCS on Farms 1 and 2 ranged from 1.5 to 5, with a mean of 2.5, and a median of 2.5 (Figure 2). Peak milk yield on Farms 1 and 2 ranged from 18 to 51 L/day, with a mean of 31.65 L/day and a median of 32 L/day (Figure 2). PPD was only recorded in 14 lactations (0.014 PPD/lactation) and included mastitis (*n* = 4), displaced abomasum (*n* = 3), pyrexia of unknown origin (*n* = 1), and late onset uterine infection (*n* = 6).

There was median 1 and 2 (95 % range 0–9 and 0–8) repeat inseminations in lactations with and without ARTD, respectively, which was not a statistically significant difference (Kruskal-Wallace *X*^2^ = 0.67, *df* = 1, *P* = 0.412).

### Abnormal Reproductive Tract Discharge

The incidence risk of ARTD/lactation recorded from all three farms was 0.16/lactation (Table 2). The incidence risk of ARTD in lactations on each farm was 0.18, 0.11, and 0.16 for Farms 1, 2, and 3, respectively, these differences were not statistically significant (*X*^2^ = 3.37, *df* = 2, *P* = 0.19).

**Table 2:** Incidence of abnormal reproductive tract discharge (ARTD) in cows and post-calving monitored periods in a study to identify the influence of ARTD and other potential risk factors on calving to conception interval in a study of dairy cows on three farms in the Riverina, NSW, Australia.

		**Incidence risk of ARTD (95% confidence interval)**			
**Denominator**	**Measure of incidence**	**Farm 1**	**Farm 2**	**Farm 3**	**Total**
lactations	Risk	0.18 (0.14–0.23)	0.11 (0.07–0.18)	0.16 (0.13–0.20)	0.16 (0.14–0.19)
Cow	Risk	0.27 (0.21–0.33)	0.16 (0.10–0.26)	0.20 (0.16–0.25)	0.22 (0.19–0.25)

The incidence risk of cows with ARTD in at least one lactation from all three farms was 0.22 (Table 2). The incidence risk of cows with ARTD on each farm was 0.27, 0.16, and 0.20 for Farms 1, 2, and 3, respectively, these differences were not statistically significant (*X*^2^ = 5.41, *df* = 2, *P* = 0.07).

The median number of examinations for cows with ARTD was 3 and was significantly greater than the median number of examinations for cows without ARTD difference (Kruskal-Wallace *X*^2^ = 95.7, *df* = 1, *P* < 0.0001; range 1–13 and 1–5 for with and without ARTD, respectively).

### Survival Analysis

Of 821 lactations in which ARTD was not observed, pregnancy was reported in 614 lactations (right-censored = 207 lactations). Of the 156 lactations in which ARTD was observed, pregnancy was reported in 98 lactations (right-censored = 58 lactations). Twenty-four pregnancies occurred prior to the 50 days VWP (3.4% of all pregnancies, at median 45 days post-calving), and all were in cows in which ARTD was not recorded. Censoring appeared to be independent of ARTD in that the distributions of censoring in cows with and without ARTD were similar [median 105 and 83 days (95% range 33–339 and 33–363) in cows with and without ARTD, respectively, Kruskal-Wallis *X*^2^ = 0.43, *df* = 1, *P* = 0.49; Supplementary Figure 1].

**Figure 3:**
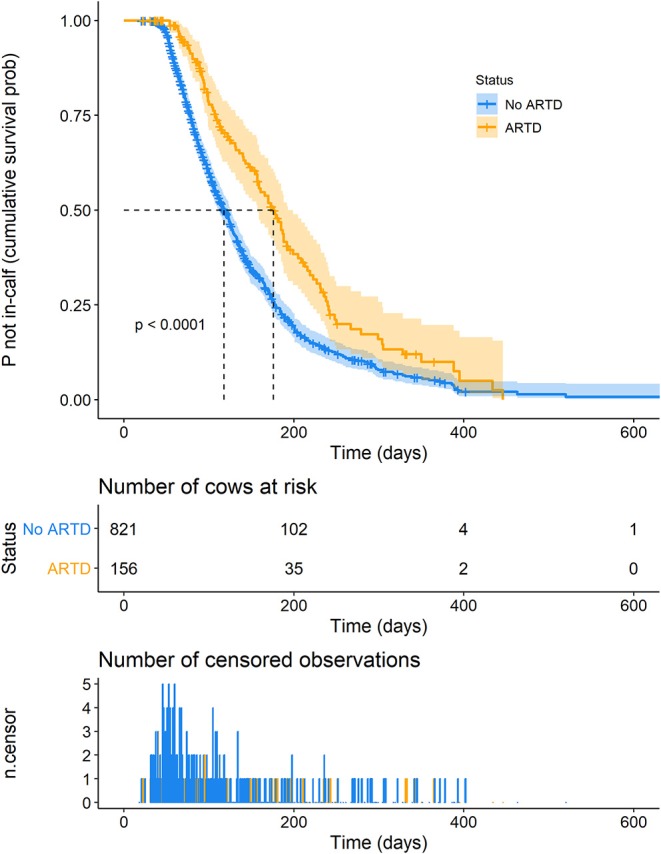
Kaplan-Meier survival curve of the probability of remaining not in-calf, dependent on the presence of abnormal reproductive tract discharge (ARTD) during lactation in a study to identify the influence of ARTD and other potential risk factors on the calving to conception interval in dairy cows on three farms in the Riverina, NSW, Australia. “Number of cows at risk” shows the number of cows that remained not in-calf at time points during lactation. “Number of censored observations” shows the number of cows that did not conceive or were not detected as having conceived during the lactation. Orange = cows with ARTD.

A plot of Kaplan-Meier survival functions of the probability of remaining not in-calf during a lactation with and without ARTD, is shown in Figure 3. The time at which 50% of cows were expected to be in-calf (the median “survival time”) was 176 days (95% CI 157–193 days) and 118 days (95% CI 112–126 days) for lactations in which the cow was diagnosed with and without ARTD, respectively (*P* < 0.0001).

### Risk Factors

Estimated total and direct (ARTD only) effects of variables of interest are shown in Table 3. ARTD significantly decreased the hazard of pregnancy (CCI was increased) approximately 0.6 times relative to the rate of pregnancy in cows without ARTD (total effect hazard ratio = 0.62, se = 0.18, *P* = 0.01). The direct and total effects of ARTD on CCI were similar, indicating that the reduced rate of pregnancy was not influenced by the indirect effects of ARTD (via influences on ovarian cyclicity; Figure 1) in this study population.

**Table 3:** The estimated total and direct [abnormal reproductive tract discharge (ARTD) only] effects of variables of interest from Farms 1 and 2 in a study to identify the influence of ARTD and other potential risk factors on the calving to conception interval in a study of dairy cows on three farms in The Riverina, New South Wales, Australia.

**Variable**	**Level**	**Effect type**	**Hazard ratio**	**se**	***P*-value**
ARTD, ref: no (*n* = 421)	Yes (*n* = 81)	Total	0.62	0.18	0.01
	Yes (*n* = 81)	Direct	0.65	0.18	0.02
BCS, ref: >4 (*n* = 131)	≤ 2 (*n* = 96)	Total	0.78	0.44	0.56
	2 ≤ 4 (264)		0.82	0.42	0.64
Parity, ref: low (≤2, *n* = 266)	High > 2 (*n* = 221)	Total	0.75	0.13	0.02
Peak milk yield, ref: low (≤ 32 L/day, *n* = 236)	High > 32 L/day (*n* = 210)	Total	0.63	0.13	<0.001
Mean maximum temperature	Continuous	Total	1.02	0.01	0.72
Peri-parturient disease, ref: no (*n* = 488)	Yes (*n* = 14)	Total	0.54	0.38	0.1

*ARTD, abnormal reproductive tract discharge; ref, reference level; se, standard error; BCS, body condition score*.

The rate of pregnancy was significantly decreased by approximately 0.6 times in cows in which the peak milk yield was >32 L/day (median peak yield) and 0.75 times in higher than median parity (>2) cows (Kaplan-Meier survivor function plots are shown in Supplementary Figures 2, 3). A significant effect of either BCS or mean daily environmental temperature was not observed in this study. The effect of periparturient disease in this study was inconclusive; although a significant effect of periparturient disease was not observed, the standard error of this effect was large, most likely due to the small number of cows with reported periparturient disease (15 cases, se = 0.38, *P* = 0.1).

Assessment of the cumulative log hazard plots of observed data indicated that the curves of the groups with and without ARTD were reasonably parallel (Supplementary Dataset 2). The curves of the hazard plots of Kaplan-Meier observed and Cox model predicted hazard plots were assessed as close (Supplementary Dataset 2). Evaluation of the change in Schoenfeld (“partial”) residuals against time for variables of interest indicated that the assumption of proportional hazards was valid for all variables (*P* > 0.1) except periparturient disease (*P* = 0.01) in both models of the direct and total effects of ARTD on CCI (Supplementary Dataset 2). Due to the few reported cases of periparturient disease, adjustments for time were not made in the Cox proportional hazard models for this variable; instead, interpretation of the effect of periparturient disease remains inconclusive in this study population.

## Discussion

The incidence of ARTD on the farms in the current study is broadly consistent with other studies in dairy herds in which an incidence of 5–43% has been estimated. This broad variation could be due to many factors such as location, management differences, diagnostic method, and definition of clinical endometritis or ARTD (22, 30, 70–73). Postpartum reproductive tract diseases in dairy cows were not clearly defined until relatively recently (74). Despite case definition differences, this broad range in incidence is still apparent when only considering studies that employed a similar definition of ARTD to the one used in this study. Previous studies that reported an incidence of ARTD close the 16% reported here include a study in Ontario, Canada, in which 16.9 % of post-calving cows had clinical endometritis (22) and a study in northeast China in which the incidence of clinical endometritis in post-calving dairy cows was 17.4% (70). In the current study, we also found that the rate of pregnancy was lower in cows in which ARTD was detected and consequently, CCI was significantly longer in lactations in which ARTD was detected—by an average of 2 months—than those in which it was not detected. Due to the factors that can influence variation in study outcomes mentioned above, and the 50 days voluntary waiting period implemented on the farms in the current study, we focus on the relative difference in CCI between cows with and without ARTD in the current study rather than absolute values of CCI for each group.

Although the association between ARTD, pregnancy rate, and prolonged CCI appears to be consistent with other studies, the magnitude of the effect of ARTD on pregnancy rate and CCI in the current study was greater than expected given the findings of other studies. When accounting for herd, parity, and ovarian status, cows diagnosed with clinical endometritis in a study in Canada had a reduced relative pregnancy rate of 27% (for example, a decrease in 21-day pregnancy rate from 20 to 14.6%), and a 32 days increase in median time to pregnancy (22). In another study, the presence of purulent or mucopurulent discharge from the uterus in the weeks prior to breeding increased the median days until pregnant by 8–18 days (75). The larger effect in the current study could be due to a range of factors, including differing environments, management factors, and host factors, that could influence the severity and persistence of endometrial inflammation or lesions, or even result in the presence of permanent changes impairing endometrial glands and altering the uterine environment (76). However, another factor that must be considered is the treatment protocol for cows with endometritis (77). Globally, different treatments for endometritis are used such as intrauterine antibiotics and systemic prostaglandin injections at varying intervals postpartum (28). In the current study, the use of prostaglandin to lyse a CL and promote oestrus in which ARTD due to endometritis could be resolved would have increased the CCI whilst waiting for the subsequent dioestrus period to confirm resolution of ARTD prior to insemination in the next oestrus. Nevertheless, this would have only increased CCI by approximately 20 days (the duration of two follicular waves) which still leaves a substantially longer CCI than expected relative to previous studies. We therefore suggest that resolution of ARTD was relatively slow in cows in the current study. This is supported by the finding that the median and upper range of the number of examinations of cows with ARTD was significantly greater than those without ARTD.

As described in the introduction, there are many factors involved in the occurrence of endometritis and prolonged CCI. Many relate to management of cows in the dry and peri-partum periods and it is possible that environmental conditions in Australia (phases of drought in many locations throughout the study period, including the Riverina, NSW) are making it more difficult to maintain metabolically healthy pasture-based dairy cows in these periods. This could be resulting in increased difficulty to resolve problems such as ARTD post-calving. Further research is needed to determine if alternate protocols to resolve ARTD would be of greater benefit on the farms in the current study, and determine the influence of dry cow and calving cow management. It would also be of interest to extend this study to further dairy farms in Australia. An economic analysis of the benefit of treatment of cows with ARTD would be warranted if our findings are reproduced. The economic benefit of treatments for endometritis have been found to be herd-specific (28), but in Australia, the largest benefit might be in management of cows to prevent ARTD and enable cows to resolve ARTD quickly rather than focusing on treatment of ARTD that is potentially difficult to resolve.

We found that CCI was also influenced by parity and peak milk yield. Consistent with these findings, Hillers et al. (46) found that cows with ≥3 parities had reduced reproductive performance compared with those of lower parities and both this latter study and Inchaisri et al. (78) found that high levels of milk production reduced reproductive performance. This is likely due to cows with higher metabolic demands needing to mobilize more adipose tissue to support lactation, thereby predisposing cows to negative health events (79, 80) and loss of reproductive success (81, 82). Hence, targeted post-calving examination of high yielding, older dairy cows might be beneficial to detect endometritis earlier. In contrast to previous studies, we did not find an association between BCS and CCI (83, 84). It has been demonstrated that cows that lost more BCS during the dry period suffered more adverse uterine health events (85). Also, cows exhibiting greater fat mobilization during the periparturient period are at a higher risk of clinical and subclinical endometritis (86). Hence, monitoring of BCS change throughout the periparturient period might have better reflected energy balance and the subsequent effects on CCI. This was not possible given the retrospective nature of our study and that BCS data prior to veterinary reproductive control visits were not available. The lack of association observed between ambient temperature and CCI in the current study is also inconsistent with the literature. Low oestrus expression and a lower conception rate usually occur during the hot season, particularly when ambient temperature is ≥30°C (45, 54). This difference might be explained by the ability of dairy farmers involved in the current study to manage dairy cows in the heat; for example, by providing shade, water, and sprinkler systems, as well as the relatively low humidity in the Riverina area.

There are some limitations associated with the current study. Detailed information about predictors that vary over time, such as BCS and milk yield throughout lactation, were not available. In future studies we suggest the inclusion of such information in the Cox proportional hazard models as time varying covariates (including ambient temperature) to determine more accurately their effect on CCI. In particular, lactation curves could be inferred from milk records (monthly on the farms in this study) so that peak yield could be more accurately defined. Early conception is not possible to measure and if embryonic death (ED) or early fetal loss (EFL) occurred prior to pregnancy diagnosis the impact of endometritis on the CCI might have been overestimated. However, we also made the assumption that ED or EFL had causes other than endometritis; if endometritis was the cause, the impact of endometritis on CCI might have been underestimated (87). In addition, the impact of genetic effects was not measured. A study of Friesian dairy cows in the United Kingdom found that commencement of luteal activity postpartum is heritable (h^2^ = 0.26; *P* = <0.001) (88) and it is possible that genetic effects were influential in the current study. Lastly, this study was on three farms in New South Wales, Australia. The size of the observed effects might be modified by factors on other farms and although we expect that our findings should be broadly generalizable to other farms with similar management systems and environments, further research is needed to validate this.

Overall, the high proportion of ARTD in dairy herds found in this study and its significant influence on the CCI reinforces the importance of reproductive management of dairy cows in the pre- and periparturient period. As well as research into the treatment and prevention of endometritis, further research on farms in other areas of Australia to determine the impact of ARTD is worthwhile because the economic impact on the dairy industry might be large. The magnitude of difference in CCI between cows with and without ARTD despite regular veterinary examination of post-calving cows brings into question the value of veterinary interventions to improve herd fertility in a herd health management program.

## Data Availability Statement

The datasets generated for this study are available on request to the corresponding author.

## Ethics Statement

The study was exempt from ethics approval by the Animal Care and Ethics Committee of Charles Sturt University because pre-existing clinical records were used.

## Author Contributions

MH collected the data. MH and VB performed the analysis. All authors conceived, designed the study, and contributed to the manuscript.

### Conflict of Interest

The authors declare that the research was conducted in the absence of any commercial or financial relationships that could be construed as a potential conflict of interest.

## Supplementary Material

Supplementary Dataset 1 Literature review undertaken to inform DAG in a study to identify the influence of endometritis and other potential risk factors on the calving to conception interval in a study of dairy cows on three farms in The Riverina, New South Wales, Australia.

Supplementary Dataset 2 log-cumulative hazard plots and observed vs. predicted hazard plots of cows with and without abnormal reproductive tract discharge (ARTD), and plots of Schoenfeld residuals of variables in Cox proportional hazards models to determine the association between ARTD and the calving to conception interval of cows in a study to identify the influence of ARTD and other potential risk factors on the calving to conception interval in dairy cows on three farms in the Riverina, NSW, Australia.

Supplementary Figure 1 Boxplots of time to right censoring in cows with and without abnormal reproductive tract discharge (ARTD) in a study to identify the influence of ARTD and other potential risk factors on the calving to conception interval in a study of dairy cows on three farms in the Riverina, New South Wales, Australia.

Supplementary Figure 2 Kaplan-Meier survival curve of the probability of remaining not in-calf dependent on peak milk yield. The table of “Number of cows at risk” shows the number of cows that remained non-pregnant at time points following calving. The graph of the “Number of censored cows” shows the number of cows that did not conceive or were not detected as having conceived in the allocated study period (orange = cows with low peak milk yield).

Supplementary Figure 3 Kaplan-Meier survival curve of the probability of remaining not in-calf dependent on parity. The table of “Number of cows at risk” shows the number of cows that remained non-pregnant at time points following calving. The graph of the “Number of censored cows” shows the number of cows that did not conceive or were not detected as having conceived in the allocated study period (orange = cows with low peak milk yield).

## Abbreviations

CCI: calving-to-conception interval
RFM: retained fetal membranes
DIM: days in milk
ARTD: abnormal reproductive tract discharge
BCS: body condition score
CI: confidence interval.
