# Supplementary material for: The Effect of Abnormal Reproductive Tract Discharge on the Calving to Conception Interval of Dairy Cows
Source: Front Vet Sci. 2019 Oct 22;6:374. doi: 10.3389/fvets.2019.00374 (PMC6817506; doi:10.3389/fvets.2019.00374)
Supplement: Supplementary Dataset 1 — Literature review undertaken to inform DAG in a study to identify the influence of endometritis and other potential risk factors on the calving to conception interval in a study of dairy cows on three farms in The Riverina, New South Wales, Australia. [file Data_Sheet_1.docx]

**Literature Review**

# Glossary of abbreviations and terms

CCI Calving to conception interval

ARTD Abnormal reproductive tract discharge

BCS Body condition score

CI Confidence interval

X^2^ Chi squared

df Degrees of freedom

se Standard error

PCMP Post-calving monitored period

LH Luteinising hormone

AI Artificial insemination

DIM Days in milk

LPS Lipopolysaccharides

TNFα Tumour necrosis factor-α

CL Corpus luteum

DAG Directed acyclic graph

ED Embryonic death

L Litres

Table of Contents

[Glossary of abbreviations and terms 2](#_Toc13080708)

[1.1 The importance of uterine health 3](#_Toc13080709)

[1.2 The importance of managing the calving to conception interval 4](#_Toc13080710)

[1.2.1 Economics 4](#_Toc13080711)

[1.2.2 Welfare 4](#_Toc13080712)

[1.3 Risk factors for endometritis and an extended calving to conception interval 5](#_Toc13080713)

[1.3.1 Host factors increasing the risk of an extended CCI and endometritis 5](#_Toc13080714)

[1.3.2 Environmental factors increasing the risk of an extended CCI and endometritis 9](#_Toc13080715)

[1.3.3 Pathogenic factors increasing the risk of endometritis 12](#_Toc13080716)

[1.4 Endometritis: definitions and diagnostics 12](#_Toc13080717)

[1.4.1 Definitions 12](#_Toc13080718)

[1.4.2 Diagnostics 13](#_Toc13080719)

[1.5 The impact of endometritis on reproduction 14](#_Toc13080720)

[1.5.1 Endometritis extending the CCI specifically 14](#_Toc13080721)

[1.5.2 Endometritis impacting ovulation and oestrus expression 15](#_Toc13080722)

[1.5.3 Endometritis impacting on conception rate and insemination number 15](#_Toc13080723)

[1.5.4 The mechanisms by which endometritis negatively impacts reproductive performance 15](#_Toc13080724)

[1.6 References 17](#_Toc13080725)

## The importance of uterine health

Postpartum uterine health is a critical factor when considering the fertility of dairy cows (1-3). Uterine involution and clearance of bacteria are key factors in achieving uterine health post-calving. Uterine involution involves the decrease of uterine size and the re-establishment of the endometrium for future pregnancies (3), and is usually completed within 40-50 days postpartum (4). Delayed uterine involution is associated with reduced fertility in dairy cows (2, 3). Uterine contamination (often difficult to distinguish from uterine infection in the field) is the presence of bacteria within the uterus without active proliferation, and does not on its own have a significant effect on reproduction (5, 6). Uterine infection can cause delayed involution and is associated with subfertility, due to perturbation of the hypothalamus-pituitary-ovarian axis. Uterine infections also directly affect the uterus, through damage to the endometrium, resulting in an environment unsuitable for the survival and growth of an embryo or fetus (1). In an active uterine infection, bacterial components (such as lipopolysaccharides) and inflammatory mediators (such as cytokines and nitric oxide) present are detrimental to the ovaries, the uterus, embryos and to the structure and function of sperm (7). Postpartum uterine health is therefore an important area for consideration in the dairy industry.

## The importance of managing the calving to conception interval

Dairy farmers around the world attempt to minimise the interval between each cow’s sequential calving, or the ‘inter-calving interval’ (ICI). This strategy increases reproductive efficiency and milk production, and ultimately optimises economic gain (8, 9). The ICI consists of the gestation period and the ‘calving to conception interval’ (CCI), which is the period between parturition and the following conception. Due to the typically fixed nature of the gestation length, the main focus is minimising the CCI. A CCI will be increased by any of the following scenarios: delayed postpartum ovulation; delayed or suppressed oestrus expression and heat detection (in herds using AI); reduced conception rates; and/or increased number of inseminations per conception (8, 10). The two main factors influenced by changes in CCI are economics and welfare.

### Economics

Minimising the CCI of dairy cows has been found to be economically beneficial (8-15). Reasons for the increased costs associated with an increased CCI include: reduced milk yields consequently lowering profit (relative to labour and feed costs) (9-11, 14, 16-18); fewer calves for sale or as replacement heifers (11, 14); reduced number of lifetime productive days (12); increased breeding expenses (through insemination, therapeutics and labour costs) (8, 11, 12, 15, 19); and higher culling rates (10-12, 19).

Conversely, some studies have found that extended calving intervals can be associated with milk yields maintained above their dry-off threshold (20) or even increased compared with shorter or average calving intervals (21). However, both of these studies focused on production levels and lacked a complete economic analysis that accounted for all potential variables. Furthermore, there is a need for studies quantifying the economic impact of CCI on farms with similar management systems to Australian dairy farms (i.e. pasture-based systems).

### Welfare

Österman, S. (21) investigated simultaneously extending CCI and increasing milking frequency. Not only did the extended CCI (both with or without increased milking frequency) increase milk yields, it was speculated that an increased milking frequency would improve the comfort of cows (21). The concept that increased milking frequency improves cow welfare is supported by Gleeson, D. E., B. O'Brien, L. BoyleandB. Earley (22). An extended calving interval would also mean fewer risk periods for each cow (for example, parturition and periparturient disease), which would increase the welfare and longevity of the cows (21). These outcomes could be economically and ethically beneficial, however a complete financial analysis is required to confirm economic feasibility.

To effectively manage the CCI, all risk factors that influence CCI must be considered.

## Risk factors for endometritis and an extended calving to conception interval

Many factors contribute to reproductive performance and ultimately to the CCI (18, 23). The CCI can be increased via delayed ovulation, delayed or suppressed oestrus, and reduced conception rates. All these events have contributing factors of their own, which ultimately extend the CCI.

CCI and endometritis share many of the same risk factors, and endometritis is a risk factor for an extended CCI. If endometritis negatively impacts on reproductive performance, then recognising and minimising the risk factors of endometritis is essential. The risk factors of endometritis are not causal alone, however they may increase the prevalence and/or severity of the disease (24).

### Host factors increasing the risk of an extended CCI and endometritis

#### Periparturient disease affecting CCI

Periparturient diseases (such as hypocalcaemia, ketosis, retained fetal membranes [RFM], endometritis, mastitis, pneumonia, lameness, and cystic ovarian disease) have been found to significantly extend the CCI (25-27). These conditions impact on the calving to first service interval, services per conception, and/or number of days open (25, 27). Prevention and management of disease in the periparturient period is vital in minimising the CCI. For more detail on endometritis as a risk factor for an extended CCI, refer to section 2.5.

#### Periparturient disease as a risk factor for endometritis

Having RFM is a major risk factor for endometritis (6, 28-34). The residual tissue creates a favourable medium for bacterial growth in the uterus, and may result in a patent cervix allowing further bacterial contamination (6, 28). Associated tissue necrosis can then cause delayed uterine involution and subsequent infection (6, 28). Metritis is also a risk factor for endometritis (35-37). The higher the degree of metritis (e.g. puerperal metritis) the higher the risk of endometritis (38). An association has been found between hypocalcaemia and a higher incidence and severity of endometritis (39, 40). Hypocalcaemia can result in complications such as reduced uterine contractions during parturition (leading to dystocia) and/or delayed uterine involution postpartum (slowing elimination of bacterial contamination), which are both risk factors for endometritis (1, 41, 42).

Retained fetal membranes, metritis, and hypocalcaemia are all periparturient conditions that elevate the risk of endometritis in dairy cows.

#### Mastitis affecting CCI

Mastitis in dairy cows has a negative impact on reproductive performance (43). A large epidemiological study (n = 222 farms, 151481 cows) found that acute clinical mastitis has a time-dependent impact on conception rate; conception rate lowers 23 & 24% if the mastitis occurs 10 days before or 30 days after artificial insemination respectively (44). Furthermore, subclinical cases (>150, 000 cells/mL) had a reduction in conception rate of 8%, and chronic subclinical cases had a reduction of 14.5-20.5% (44).

#### Mastitis as a risk factor for endometritis

A study by Bacha, B.andF. G. Regassa (45) found a positive relationship between mastitis and subclinical endometritis. Endometritis and mastitis share certain potential risk factors such as RFM, hypocalcaemia, multiparity, and calving during winter (42). Therefore, there are confounding factors to consider when determining a relationship between mastitis and endometritis (42). Potter, T. J., J. Guitian, J. Fishwick, P. J. GordonandI. M. Sheldon (6) found a positive, but insignificant (p = 0.225), association between mastitis and clinical endometritis.

#### Body condition affecting CCI

Body condition can be measured using a ‘Body Condition Score’ (BCS) on a scale of 1 – 5 that reflects over or underweight body condition (46). Both high (>3) and low (<3) BCS are associated with lower conception rates and prolonged onset of oestrus (47-49). A reduction in BCS during the postpartum period (a loss of at least 1.25 BCS units) reduces the likelihood of conception to first service, prolongs calving to conception intervals, and increases the number of inseminations required per conception (26, 50). Overall, maintaining a BCS that is not too high or low (BCS ~3) in the periparturient period is important to prevent extension of the CCI.

#### Body condition as a risk factor for endometritis

A low BCS, or reduction in BCS, in the periparturient period is also a significant risk factor for endometritis (37, 41, 45, 51-53). LeBlanc, S. J. (54) found that a high BCS may also lead to endometritis.

#### Negative energy balance affecting CCI

In the early postparturient period, dairy cows undergo extensive tissue catabolism, especially if in a negative nutrient balance (55). Uncontrolled metabolic imbalances can lead to metabolic diseases, which considerably reduce reproductive performance (41, 55). A negative nutrient balance involves low circulating concentrations of insulin and glucose as well as higher concentrations of non-esterified fatty-acids and ketone bodies, which have detrimental effects on oocytes and on the pattern of ovarian follicular growth (55). Cows in a negative nutrient balance have reduced pulse frequency of luteinising hormone (LH) and prolonged periods of anovulation and anoestrous, due to disrupted metabolic signals and regulatory hormones (41, 55, 56). A negative energy balance is also associated with a higher risk of uterine disease, which impairs reproductive efficiency (41, 55, 56).

#### Negative energy balance as a risk factor for endometritis

Negative energy balance in the postpartum period can lead to a dysregulated immune response (57), inflammation including that of the uterus, and delayed uterine involution. All of which increase the likelihood of endometritis (24, 40, 56). A negative energy balance can also lead to many metabolic conditions such as ketosis (24, 40). Ketosis is a risk factor for both subclinical endometritis (35, 37) and clinical endometritis (28, 36, 38). Reducing the degree of negative energy balance by improving nutritional management may be important in reducing endometritis in dairy herds.

#### Parity affecting CCI

Nulliparous (parity ‘0’) heifers have higher conception rates than lactating cows (58). Cows of third or higher parities have reduced reproductive performance compared with those of lower parities (59, 60), and primiparous (first-calving) cows have prolonged involution and increased days between calving and ovulation compared with multiparous cows (60). In summary, nulliparous (parity ‘0’) and second-parity cows appear to have the highest reproductive success.

#### Parity as a risk factor for endometritis

Findings regarding the relationship between parity and endometritis in dairy cows are inconsistent (24). Some studies found no association between parity and endometritis (38, 51). In others, a higher prevalence of endometritis was found among primiparous groups, possibly due to their elevated risk of dystocia, endometrial lesions, and infection with *Trueperella pyogenes* (6, 30, 42, 52, 61). However, Kim, I. H.andH. G. Kang (28) found endometritis to be less common in primiparous cows due to faster uterine involution compared with multiparous cows. In some reports higher parity has been found to be a risk factor of clinical endometritis (31, 62). Older cows have less uterine elasticity and slower uterine involution, which may lead to more persistent infections (24). Alternatively, higher-parity cows may have had more exposure to bacterial contamination and therefore have an enhanced immunological response allowing them to eliminate uterine bacteria more efficiently (24). The balance of risk factors and protective mechanisms might explain the varied results regarding parity as a risk factor of endometritis (24).

#### Milk yield affecting CCI

Milk production influences the reproductive performance of dairy cows and the subsequent CCI (59, 63). There is a trend between higher milk yields in the current or previous lactation and a prolonged interval to first service (59). A high milk yield at the time of first insemination has been found to reduce first insemination success rates (63). In short, there is a difficult balance between achieving high production levels without compromising reproductive performance, likely due to the increased nutrient demands associated with an increased milk yield (see section on ‘Nutrition affecting CCI’ below).

#### Genetic predisposition and CCI

A common question discussed in the literature is to what extent genes play a role in the reproductive performance of dairy cows. Cows with a higher genetic potential for milk production have frequently been shown to have worse reproductive performance (23). Favourable alleles for milk production often differ from the favourable alleles for reproduction (64). Interestingly, four genetic reproductive trait loci have been identified that affect reproduction without affecting production (65) and certain variants have been associated with calving efficiency (66). However, the heritability of calving interval was calculated to be only 0.01 (67, 68), and when dealing with the conflicting genetic traits of production and reproduction, the effect on reproduction was relatively small (64).

Significant differences in fertility and calving interval have been found between different breeds of cattle, but less so within breed (64, 67, 69, 70). In the present study, the impact of genetic variation was not included as a potential risk factor, because all cows were the same breed and were all artificially inseminated from a relatively small semen pool.

#### Dystocia as a risk factor for endometritis

Many studies have found dystocia (30, 33, 34, 36-38) and calving assistance (6, 71) to significantly increase the risk of endometritis. Dystocia has the potential to cause endometrial trauma, and calving assistance increases the risk of introducing bacteria into the uterus (30). Furthermore, dystocia is associated with postpartum complications such as RFM, metritis and delayed uterine involution, all of which are risk factors of endometritis (42, 72, 73). Male fetuses, which are often larger than females, increase the risk of dystocia and therefore increase the risk of endometritis (6, 24, 42). Dystocia is therefore a significant and important risk factor of endometritis.

#### Cyclicity as a risk factor for endometritis

The resumption of ovarian activity postpartum positively influences uterine involution and therefore has the potential to reduce the risk of endometritis (24, 74). Mee, J. F. (40) found delayed ovulation to be a risk factor for endometritis. However Sheldon, I. M.andH. Dobson (1) did not find a significant impact of ovarian cyclicity on endometritis, and Carneiro, L. C., A. F. Ferreira, M. Padua, J. P. Saut, A. S. FerraudoandR. M. Dos Santos (51) found that endometritis was not influenced by the presence of a corpus luteum.

### Environmental factors increasing the risk of an extended CCI and endometritis

#### Season / Ambient temperature affecting CCI

Insemination of dairy cows during hotter seasons results in reduced oestrus expression and conception rates (58, 75). There is a dramatic decline in the conception rate of lactating cows (a reduction of 20%) when maximum ambient temperature exceeds 30˚C on the day after insemination (58). Interestingly, the conception rate of heifers does not begin to decline until the ambient temperature reaches 35˚C (58). Heat stress modifying the uterine environment and reducing the chance of embryo attachment could be a mechanism by which this occurs (75). Therefore, management of postpartum cows in hot weather (for example with cooling systems involving shade, fans, and/or sprinklers) may be warranted to optimise reproductive performance (58, 76).

#### Season as a risk factor for endometritis

Results vary as to whether calving season increases the risk of endometritis (24). Bruun, J., A. K. ErsbøllandL. Alban (30) and Ghavi Hossein-Zadeh, N.andM. Ardalan (42) both found an increase in metritis (a risk factor for endometritis) when cows were calving in the winter months. In another study, primiparous cows reportedly had higher levels of metritis in cooler seasons, however season had no effect on multiparous cows (34). Kim, I. H.andH. G. Kang (28) and Carneiro, L. C., A. F. Ferreira, M. Padua, J. P. Saut, A. S. FerraudoandR. M. Dos Santos (51) found no association between calving season and endometritis.

#### Nutrition affecting CCI

Inadequate nutritional management of dairy cows, especially in the periparturient period, is a key driver of poor reproductive performance (41). The nutritional management of dairy cows in pasture-based systems is reviewed by Butler, S. T. (77).

Conception rates were found to be higher, CCIs shorter, and oestrus expression earlier and more noticeable in cows with higher dry matter intakes, increased glucose:hydroxybutyrate ratios, and

higher plasma cholesterol concentrations (78). Too much feed and/or particular nutrients in excessive quantities can, however, increase the metabolic clearance of steroid hormones, such as oestradiol and progesterone, which may cause a suboestrous, reducing oestrus detection (41). Moderate quantities of supplementary fat in the diet can increase caloric intake, improve ovarian dynamics, regulate prostaglandin secretion from the uterus, optimise luteal function, and have a positive effect on fertility (55). However, excessive levels of non-esterified fatty acids have been associated with lower conception rates (78). Excess protein intake, particularly diets high in rumen-degradeable-protein, can reduce first-service conception rates and lengthen calving to conception intervals (55, 78).

Nutrition in the periparturient period has a major impact on reproductive performance by affecting oocyte viability, ovulation, oestrus expression, and conception rates, which all impact the CCI.

#### Nutrition as a risk factor for endometritis

The quantity and quality of protein, vitamins, and minerals in a dairy cow diet can have an impact on the occurrence of endometritis (24). A deficiency or excess of protein in the diet can affect the immune system and be a risk factor for endometritis (24, 79, 80). Vitamins such as vitamin A, B, C, and E are important for the immune system (24, 81). Vitamin A also has a role in epithelial tissue development, which is beneficial for healing any reproductive tract trauma in the postpartum period (24, 82). A number of minerals assist in uterine defense mechanisms (24). Calcium, for example, is a complement activator, and deficiencies delay uterine involution and prolong uterine infections (24, 83). Selenium assists with the function of neutrophils and deficiencies of selenium disrupt reproductive performance and increase the risk of endometritis (84). Magnesium facilitates opsonisation mechanisms, which helps immune cells with phagocytosis (79). Copper, zinc, and iron are also beneficial for phagocytosis, and deficiencies allow increased bacterial growth and raise the risk of endometritis (79, 83). Furthermore, imbalances in vitamins and minerals can be detrimental to reproductive performance in an unspecific manner (83).

Nutrition is therefore an important factor influencing the occurrence of endometritis.

#### Heat detection and AI technique affecting CCI

Artificial insemination (AI) technique and oestrus-detection method or ability varies between technicians and has an influence on reproductive measures such as the CCI (49, 58, 85, 86). Conception rates of dairy cows vary between AI technicians (with rates differing by up to 15%) (49).

Inadequate ability to detect oestrus has a strong association with an increased calving interval (86). Signs chosen to detect oestrus also has an influence on CCI (49, 85). For example, inseminations based on mounting activity, genital discharge, and restlessness and/or other signs, obtained first-service conception rates of 53.6, 48.8 and 50.1%, respectively (p < 0.05) (49). Oestrus-detection timing and routines is another influential factor on CCI (86). For example, oestrus-detection during feeding time was associated with an increased calving interval (86). This is thought to be because the staff’s attention is divided during this time. Reproductive efficiency can be improved on dairy farms through training staff in reproductive management (49).

#### Environmental cleanliness as a risk factor for endometritis

Risk factors of endometritis tend not to be associated with environmental contamination, but rather with trauma of the reproductive tract and disruption of physical barriers to infection (6). Williams, E. J., D. P. Fischer, D. U. Pfeiffer, G. C. W. England, D. E. Noakes, H. DobsonandI. M. Sheldon (5) found that clinical endometritis was associated with the density of pathogenic bacteria, but not of opportunistic contaminants. It appears as though environmental contamination does not have a significant influence on the occurrence of endometritis.

### Pathogenic factors increasing the risk of endometritis

In the first two weeks postpartum, at least 90% of dairy cows have bacterial contamination in the uterus (5, 87-89). Although cows usually eliminate these bacteria over the next five weeks, contamination transitions to infection in over 10% of cows, and endometritis develops (5, 87, 90). There are many bacteria that can be present in the uterus of cows with endometritis (24, 71). *Trueperella pyogenes* and *Escherichia coli* are the most common bacteria isolated from the uterine lumen in cows with an infected uterus, followed by anaerobic bacteria including *Prevotella* spp. and *Fusobacterium nucleatum* (24, 61, 71, 91-93).

## Endometritis: definitions and diagnostics

### Definitions

The term ‘endometritis’ is often used incorrectly to include pyometra, metritis, and endometritis (91), and endometritis and metritis are often used interchangeably (94). Pyometra is an accumulation of purulent material in the lumen of a uterus, with a closed cervix and a persistent corpus luteum (91). Metritis is pathologically defined as inflammation of the full thickness of the uterine wall (95), and in a field setting is defined as a condition occurring within 21 days of parturition, involving an enlarged uterus and fetid purulent uterine discharge in the vagina (24, 91). Puerperal metritis is a branch of metritis in which the cow also has systemic signs (e.g. hypo- or hyperthermia, tachycardia, tachypnoea, decreased milk yield, and anorexia), and usually occurs within 10 days of parturition (24, 91, 96).

Endometritis is defined as inflammation of the endometrium alone (95) and is often classified as clinical or subclinical (91). In the field, diagnosis of clinical endometritis involves detecting purulent (>50% pus) uterine discharge in the vagina ≥21 days postpartum, or mucopurulent (approximately 50% mucus, 50% pus) uterine discharge in the vagina when >26 days postpartum (1, 24, 91, 94). Cases of abnormal discharge that occur <21 days postpartum are not classified as clinical endometritis because the varying appearances of normal lochia would interfere with the diagnosis, and because they will include many cows that are spontaneously recovering from normal postpartum bacterial contamination (91). Madoz, L. V., M. J. Giuliodori, A. L. Migliorisi, M. JaureguiberryandR. L. de la Sota (93) define subclinical endometritis as having ≥8% neutrophils in uterine cytology samples (collected using a cytobrush) between days 21-33 postpartum, having ≥6% neutrophils between 34-47 days or ≥4% after 47 days postpartum. However, there is a lack of agreement in the literature about the normal neutrophil percentage at increasing days postpartum (97). Dubuc, J., T. F. Duffield, K. E. Leslie, J. S. WaltonandS. J. Leblanc (98) suggested that cytological and clinical endometritis may be different manifestations of reproductive tract disease and that the term clinical ‘endometritis’ may not be accurate. Consequently, ‘purulent vaginal discharge’ may be a more accurate description.

This study will focus on postpartum clinical endometritis, according to the definitions above.

### Diagnostics

The dairy industry requires a practical method of diagnosing clinical endometritis using criteria that distinguish cows that will have compromised reproductive performance, to establish a prognosis and allow for timely treatment (91, 94). Current diagnostic methods for endometritis include manual vaginal examination, vaginoscopy, hysteroscopy, Metricheck® (Simcro, NewZealand), ultrasonography, transrectal palpation, cytology, histopathology, and diagnostic procedures such as leukocyte esterase assessment (94, 99-102). Unfortunately, there is no ‘gold standard’ method to diagnose uterine disease, leading to difficulties in measuring their sensitivity, specificity, efficiency, and overall accuracy (91). Histopathological analysis of endometrial biopsies is said to be definitive for endometritis and predictive of subsequent reproductive performance (91, 100); however, this technique is expensive, time-consuming, usually impractical, and may impair fertility (91). Cytology is a less invasive way of diagnosing subclinical endometritis; however, neither histopathology nor cytology allows for a rapid diagnosis, so clinical examination is often used to diagnose endometritis in the field (91, 103).

Transrectal palpation of the uterus is the most common method used to diagnose endometritis (94). However, this technique is subjective and does not usually consider normal processes and the variability of uterine involution, and assessments have not been shown to be associated with reproductive performance (94, 104). Rectal uterine palpation alone as diagnosis for endometritis is inaccurate, leading to many false positives and negatives (94).

The most useful method of diagnosing clinical endometritis in the field is to examine the vagina for reproductive tract discharge (91, 94, 103, 105). A study by Rahman, M. A., M. G. S. AlamandM. Shamsuddin (102) compared different levels of vaginal discharge to endometrial histopathology changes. The agreement between vaginal discharge and biopsy score was regarded as ‘good’ (102). One potential limitation is that vaginal examination might result in false positive diagnoses due to non-uterine inflammation (91, 98, 100, 106, 107). A diagnosis can be achieved by inspecting the vagina for the presence of discharge that is purulent or malodorous when ≥21 days in milk (DIM) or mucopurulent discharge when >26 DIM (91, 94).

Methods to identify this discharge include vaginoscopy (91, 94, 108), use of devices such as the Metricheck^®^, and manual vaginal examination (91). A positive vaginoscopy score is associated with lower fertility (109, 110). Factors such as the cost, inconvenience and an overestimated potential for disease transmission have reduced how commonly vaginoscopes are used (91, 94). Metricheck^®^ devices have been used to efficiently assess vaginal contents (91). A positive Metricheck^®^ diagnosis of endometritis has been associated with reduced reproductive performance (99). Although the Metricheck^®^ had a higher sensitivity than vaginoscopy, specificity was lower, leading to more false positives and a poorer positive predictive value (99). Any of these three techniques can be used to diagnose clinical endometritis without negatively affecting reproductive performance (91, 101).

The simplest technique to examine the vagina for abnormal discharge is to manually examine the vagina through the vulva (after cleaning the vulva with dry paper towel) with a clean, lubricated, gloved hand and to withdraw the contents for inspection (91, 111). This technique does not cause bacterial contamination of the uterus, delayed uterine involution, or instigate an acute phase protein response (111). This was the method used to diagnose clinical endometritis in the present study.

## The impact of endometritis on reproduction

Many studies have demonstrated the negative impact of endometritis on the reproductive performance of dairy cows (24, 112, 113). As previously mentioned, the CCI can be extended in several ways including delayed postpartum ovulation, delayed or suppressed oestrus expression and detection, reduced conception rates, and/or an increased number of inseminations per conception. The effect of endometritis on these categories, and ultimately on CCI, is discussed below.

### Endometritis extending the CCI specifically

Postpartum dairy cows with clinical or subclinical endometritis have been found to have poor reproductive efficiency, an extended CCI, and a higher risk of culling (38, 112, 114). Clinical endometritis has a higher impact than subclinical endometritis, but the negative effects on reproduction of clinical and subclinical endometritis were found to be additive (37, 112). Cows with clinical and subclinical endometritis had an increase in CCI of 57 days and 42 days, respectively, compared to those without clinical and subclinical endometritis (112). Giuliodori, M. J., R. P. Magnasco, D. Becu-Villalobos, I. M. Lacau-Mengido, C. A. RiscoandR. L. de la Sota (38) found that clinical endometritis extended the CCI by 70 days, and Barański, W., M. Podhalicz-Dzięgielewska, S. ZduńczykandT. Janowski (115) found that subclinical endometritis to increased the CCI by 30 days.

### Endometritis impacting ovulation and oestrus expression

A study on the effects of subclinical endometritis demonstrated a longer interval (by 13 days) between calving and ovulation in cows with subclinical endometritis compared with those without (116). Studies investigating clinical endometritis have found a delayed postpartum resumption of normal ovarian activity (113, 117) and lower oestrus expression/detection (99) in those with clinical endometritis compared with those without. However, Gobikrushanth, M., R. Salehi, D. J. AmbroseandM. G. Colazo (118) found that neither subclinical or clinical endometritis had an effect on follicular growth or ovulation. The small sample size (n = 126), and high proportion of diagnosed endometritis (58%) may have affected the results in this study (118).

### Endometritis impacting on conception rate and insemination number

Many studies have demonstrated a reduction in conception rate and an increase in required inseminations in cows with endometritis (92, 119, 120). One study revealed that cows with clinical and subclinical endometritis had 0.36 and 0.32 times the odds respectively of conceiving at the first service compared with those without endometritis (119). Others have found lower overall conception rates in cows with endometritis, for example a 14% (120) and a 25% (92) reduction in those with subclinical endometritis, and a 20% reduction in those with clinical endometritis (113). Endometritis has been associated with an

increased number of inseminations per conception. For example the average number of inseminations needed before 50% of the herd was pregnant was three for those with subclinical endometritis and two for those without (92).

### The mechanisms by which endometritis negatively impacts reproductive performance

Inflammation of the endometrium has a detrimental effect on reproductive performance through altering the uterine environment, and disrupting hormonal pathways, ovulation, conception, or embryo survival (1, 7, 74, 121). These effects are mediated by bacterial products such as lipopolysaccharides (LPS), or by inflammatory mediators such as nitric oxide, cytokines, and oxidative stress, which then affect the functionality of the hypothalamus, pituitary, ovary, uterus, and sperm (1, 7).

Absorption of bacterial by-products can impair the follicular phase LH surge, hinder ovulation, and lead to a slower growth rate of the first postpartum dominant follicle, which then reduces secreted oestradiol towards the end of the growth phase (1). LPS and tumour necrosis factor-α (TNFα) impair the steroidogenesis of ovarian granulosa cells, which are responsible for the production of oestradiol (7, 74).

The inflammatory uterine environment has negative effects on sperm function and motility, and increases sperm phagocytosis (7). If fertilisation of the oocyte does occur, the resulting zygote may be subjected to oxidative stress, LPS, and/or prostaglandin F2α causing lysis of the corpus luteum, and it is therefore less likely to reach the blastocyst stage (7). Embryos exposed to inflammatory mediators have less trophectoderm cells. Nitric oxide impacts embryo development and TNFα can cause blastomere apoptosis (7). Cows with endometritis often have lower peripheral concentrations of progesterone, which further reduces the chances of conception and maintenance of pregnancy (74).

Bacteria such as *Trueperella pyogenes* and *Escherichia coli* cause damage to the endometrium and generalised inflammation (74). As well as the effects described, this can reduce likelihood of embryo attachment (1, 74).

The extent of the negative effects of endometritis on reproduction depends on the severity of inflammation, the time until resolution of endometrial lesions, and on any permanent changes that impact endometrial gland function or alter the oviductal/uterine environment (105).

## References

1. I. M. Sheldon and H. Dobson: Postpartum uterine health in cattle. *Animal reproduction science*, 82-83, 295 (2004)

2. N. C. Buch, W. S. Tyler and L. E. Casida: Postpartum estrus and involution of the uterus in an experiemtnal herd of Holstein-Fresian cows. . *Journal of Dairy Science*, 38, 73-9 (1955)

3. G. Opsomer, P. Mijten, M. Coryn and A. D. FKruif: Post-partum anoestrus in dairy cows: a review. *Veterinary Quarterly*, 2, 68-75 (1996)

4. H. T. Gier and G. B. Marion: Uterus of the cow after parturition: Involution changes. *American Journal of Veterinary Research*, 29(1), 83-96 (1968)

5. E. J. Williams, D. P. Fischer, D. U. Pfeiffer, G. C. W. England, D. E. Noakes, H. Dobson and I. M. Sheldon: Clinical evaluation of postpartum vaginal mucus reflects uterine bacterial infection and the immune response in cattle. *Theriogenology*, 63(1), 102-17 (2005) doi:10.1016/j.theriogenology.2004.03.017

6. T. J. Potter, J. Guitian, J. Fishwick, P. J. Gordon and I. M. Sheldon: Risk factors for clinical endometritis in postpartum dairy cattle. *Theriogenology*, 74(1), 127-34 (2010) doi:10.1016/j.theriogenology.2010.01.023

7. R. O. Gilbert: The effects of endometritis on the establishment of pregnancy in cattle. *Reproduction, Fertility and Development*, 24(1), 252-257 (2012) doi:10.1071/RD11915

8. H. Ghiasi, A. Pakdel, A. Nejati-Javaremi, O. González‐Recio, M. J. Carabaño, R. Alenda and A. Sadeghi‐Sefidmazgi: Estimation of economic values for fertility, stillbirth and milk production traits in Iranian holstein dairy cows. *Iranian Journal of Applied Animal Science*, 6(4), 791-795 (2016)

9. A. M. Safiullah, R. Prabaharan and P. Sadasivam: Economic analysis of calving interval of Hungarian dairy cattle. *Journal of Applied Animal Research*, 19(2), 237-246 (2001) doi:10.1080/09712119.2001.9706729

10. C. Inchaisri, R. Jorritsma, P. L. Vos, G. C. van der Weijden and H. Hogeveen: Economic consequences of reproductive performance in dairy cattle. *Theriogenology*, 74(5), 835-46 (2010a) doi:10.1016/j.theriogenology.2010.04.008

11. V. E. Cabrera: Economics of fertility in high-yielding dairy cows on confined TMR systems. *Animal: an International Journal of Animal Bioscience*, 8(s1), 211-21 (2014) doi:10.1017/S1751731114000512

12. O. González-Recio, M. A. Pérez-Cabal and R. Alenda: Economic value of female fertility and its relationship with profit in Spanish

dairy cattle. *Journal of Dairy Science*, 87(9), 3053-3061 (2004) doi:10.3168/jds.S0022-0302(04)73438-4

13. C. Meadows, P. J. Rajala-Schultz and G. S. Frazer: A spreadsheet-based model demonstrating the nonuniform economic effects of varying reproductive performance in Ohio dairy herds. *Journal of Dairy Science*, 88(3), 1244-1254 (2005)

14. L. Ózsvári, F. Tóth, G. Gábor and O. Szenci: The economic importance of reproductive management in dairy herds. *Revista Română de Medicină Veterinară*, 17, 37-46 (2007)

15. A. R. Roodbari, H. Kohram and E. Dirandeh: Evaluating economic losses associated with delayed conception in dairy cows. *Iranian Journal of Animal Science*, 46(2), 151-158 (2015)

16. M. Abdullah, T. K. Mohanty, A. Kumaresan, A. K. Mohanty, A. R. Madkar, R. K. Baithalu and M. Bhakat: Early pregnancy diagnosis in dairy cattle: Economic importance and accuracy of ultrasonography. *Advances in Animal and Veterinary Sciences*, 2(8), 464-467 (2014) doi:10.14737/journal.aavs/2014/2.8.464.467

17. W. L. Adamowicz, F. S. Novak and J. J. Kenelly: The economic analysis of the delayed breeding of dairy cows. *Agriculture and Forestry Bulletin*, 8(4), 3-7 (1985)

18. R. D. Shanks, A. E. Freeman and P. J. Berger: Relationship of reproductive factors with interval and rate of conception. *Journal of Dairy Science*, 62(1), 74-84 (1979) doi:0.3168/jds.S0

19. D. Boichard: Estimation of the economic value of conception rate in dairy cattle. *Livestock Production Science*, 24(3), 187-204 (1990) doi:Doi 10.1016/0301-6226(90)90001-M

20. M. J. Auldist, G. O'Brien, D. Cole, K. L. Macmillan and C. Grainger: Effects of varying lactation length on milk production capacity of cows in pasture-based dairying systems. *Journal of Dairy Science*, 90(7), 3234-41 (2007) doi:10.3168/jds.2006-683

21. S. Österman: Extended calving interval and increased milking frequency in dairy cows: Effects on productivity and welfare. In: *Department of animal nutrition and management*. Swedish University of Agricultural Sciences, Uppsala, Sweden (2003)

22. D. E. Gleeson, B. O'Brien, L. Boyle and B. Earley: Effect of milking frequency and nutritional level on aspects of the health and welfare of dairy cows. *Animal*, 1(1), 125-32 (2007) doi:10.1017/S1751731107658030

23. D. Boas, E. Oliveira, D. Da Cruz, S. Silva, T. Samóra, P. Pires and L. Faro: Factors affecting the reproductive traits of Holstein cows. *Boletim de Indústria Animal*, 69, 40 (2012)

24. M. Adnane, R. Kaidi, C. Hanzen and G. C. W. England: Risk factors of clinical and subclinical endometritis in cattle: A review. *Turkish Journal of Veterinary and Animal Sciences*, 41(1), 1-11 (2017) doi:10.3906/vet-1603-63

25. S. Borsberry and H. Dobson: Periparturient diseases and their effect on reproductive performance in five dairy herds. *Veterinary Record*, 124(9), 217-219 (1989)

26. P. Gillund, O. Reksen, Y. T. Gröhn and K. Karlberg: Body condition related to ketosis and reproductive performance in Norwegian dairy cows. *Journal of Dairy Science*, 84(6), 1390-1396 (2001)

27. D. A. V. Timarán, C. J. B. Melo, D. P. M. Patiño, J. M. A. Martínez and C. A. C. Velásquez: Effect of early postpartum diseases on calving - conception interval: A cohort study in dairy cows from Pasto, Colombia. *Revista CES Medicina Veterinaria y Zootecnia*, 12(1), 33-43 (2017) doi:10.21615/

28. I. H. Kim and H. G. Kang: Risk factors for postpartum endometritis and the effect of endometritis on reproductive performance in dairy cows in Korea. *Journal of Reproduction & Development*, 49(6), 485-491 (2003)

29. S. J. LeBlanc: Postpartum uterine disease and dairy herd reproductive performance: A review. *The Veterinary Journal*, 176(1), 102-14 (2008) doi:10.1016/j.tvjl.2007.12.019

30. J. Bruun, A. K. Ersbøll and L. Alban: Risk factors for metritis in Danish dairy cows. *Preventive Veterinary Medicine*, 54(2), 179-190 (2002)

31. G. Gautam, T. Nakao, M. Yusuf and K. Koike: Prevalence of endometritis during the postpartum period and its impact on subsequent reproductive performance in two Japanese dairy herds. *Animal Reproduction Science*, 116(3-4), 175-87 (2009) doi:10.1016/j.anireprosci.2009.02.001

32. B. Salasel, A. Mokhtari and T. Taktaz: Prevalence, risk factors for and impact of subclinical endometritis in repeat breeder dairy cows. *Theriogenology*, 74(7), 1271-8 (2010) doi:10.1016/j.theriogenology.2010.05.033

33. D. Tayebwa, G. Bigirwa, J. Byaruhanga and K. Kasozi: Prevalence of endometritis and its associated risk factors in dairy cattle of Central Uganda. *American Journal of Experimental Agriculture*, 7(3), 155-162 (2015) doi:10.9734/ajea/2015/15816

34. M. E. Benzaquen, C. A. Risco, L. F. Archbald, P. Melendez, M. J. Thatcher and W. W. Thatcher: Rectal temperature, calving-related factors, and the incidence of puerperal metritis in postpartum dairy cows. *Journal of Dairy Science*, 90(6), 2804-14 (2007) doi:10.3168/jds.2006-482

35. S. H. Cheong, D. V. Nydam, K. N. Galvao, B. M. Crosier and R. O. Gilbert: Cow-level and herd-level risk factors for subclinical endometritis in lactating Holstein cows. *Journal of Dairy Science*, 94(2), 762-70 (2011) doi:10.3168/jds.2010-3439

36. M. J. Giuliodori, R. P. Magnasco, D. Becu-Villalobos, I. M. Lacau-Mengido and C. A. Risco: Clinical endometritis in dairy cows: Risk factors and reproductive performance. *Taurus*, 16(61), 25-33 (2014)

37. J. Dubuc, T. F. Duffield, K. E. Leslie, J. S. Walton and S. J. LeBlanc: Risk factors for postpartum uterine diseases in dairy cows. *Journal of Dairy Science*, 93(12), 5764-5771 (2010)

38. M. J. Giuliodori, R. P. Magnasco, D. Becu-Villalobos, I. M. Lacau-Mengido, C. A. Risco and R. L. de la Sota: Clinical endometritis in an Argentinean herd of dairy cows: risk factors and reproductive efficiency. *Journal of Dairy Science*, 96(1), 210-8 (2013) doi:10.3168/jds.2012-5682

39. L. C. Whiteford and I. M. Sheldon: Association between clinical hypocalcaemia and postpartum endometritis. *Veterinary Record*, 157(7), 202-204 (2005)

40. J. F. Mee: Risk factors for reproductive disorders (metritis, endometritis, cystic ovaries, anovulation, low conception rate, embryonic mortality) in dairy cows. *Cattle Practice*, 22(1), 42-53 (2014)

41. J. F. Roche: The effect of nutritional management of the dairy cow on reproductive efficiency. *Animal Reproduction Science*, 96(3-4), 282-296 (2006) doi:10.1016/j.anireprosci.2006.08.007

42. N. Ghavi Hossein-Zadeh and M. Ardalan: Cow-specific risk factors for retained placenta, metritis and clinical mastitis in Holstein cows. *Veterinary Research Communications*, 35(6), 345-54 (2011) doi:10.1007/s11259-011-9479-5

43. J. Olechnowicz and J. M. Jaśkowski: A connection between mastitis during early lactation and reproductive performance of dairy cows – A review. *Annals of Animal Science*, 13(3), 435–448 (2013) doi:10.2478/aoas-2013-0030

44. Y. Lavon, E. Ezra, G. Leitner and D. Wolfenson: Association of conception rate with pattern and level of somatic cell count elevation relative to time of insemination in dairy cows. *Journal of Dairy Science*, 94(9), 4538-4545 (2001)

45. B. Bacha and F. G. Regassa: Subclinical endometritis in Zebu x Friesian crossbred dairy cows: its risk factors, association with subclinical mastitis and effect on reproductive performance. *Tropical Animal Health & Production*, 42(3), 397-403 (2010) doi:10.1007/s11250-009-9433-5

46. A. J. Edmonson, I. J. Lean, L. D. Weaver, T. Farver and G. Webster: A body condition scoring chart for Holstein dairy cows. *Journal of Dairy Science*, 72(1), 68-78 (1989)

47. V. S. Machado, L. S. Caixeta, J. A. McArt and R. C. Bicalho: The effect of claw horn disruption lesions and body condition score at dry-off on survivability, reproductive performance, and milk production in the subsequent lactation. *Journal of Dairy Science*, 93(9), 4071-8 (2010) doi:10.3168/jds.2010-3177

48. J. E. Santos, H. M. Rutigliano and M. F. Sá Filho: Risk factors for resumption of postpartum estrous cycles and embryonic survival in lactating dairy cows. *Animal Reproduction Science*, 110(3-4), 207-21 (2009) doi:10.1016/j.anireprosci.2008.01.014

49. M. A. Siddiqui, Z. C. Das, J. Bhattacharjee, M. M. Rahman, M. M. Islam, M. A. Haque, J. J. Parrish and M. Shamsuddin: Factors affecting the first service conception rate of cows in smallholder dairy farms in Bangladesh. *Reproduction in Domestic Animals*, 48(3), 500-5 (2013) doi:10.1111/rda.12114

50. M. Doležalová, L. Stádník, M. Nejdlová, D. Němečková, J. Beran and J. Ducháček: The relationship between energy balance after calving and reproductive functions in Holstein dairy cows treated by the OVSYNCH system. *Acta Universitatis Agriculturae et Silviculturae Mendelianae Brunensis*, 61(3), 601-610 (2013) doi:10.11118/actaun201361030601

51. L. C. Carneiro, A. F. Ferreira, M. Padua, J. P. Saut, A. S. Ferraudo and R. M. Dos Santos: Incidence of subclinical endometritis and its effects on reproductive performance of crossbred dairy cows. *Tropical Animal Health & Production*, 46(8), 1435-9 (2014) doi:10.1007/s11250-014-0661-y

52. M. J. Giuliodori, M. Magnasco, R. P. Magnasco, I. M. Lacau-Mengido and R. L. de la Sota: Purulent vaginal discharge in grazing dairy cows: Risk factors, reproductive performance, and prostaglandin F2alpha treatment. *Journal of Dairy Science*, 100(5), 3805-3815 (2017) doi:10.3168/jds.2016-11373

53. A. Kadivar, M. R. Ahmadi and M. Vatankhah: Associations of prepartum body condition score with occurrence of clinical endometritis and resumption of postpartum ovarian activity in dairy cattle. *Tropical Animal Health & Production*, 46(1), 121-6 (2014) doi:10.1007/s11250-013-0461-9

54. S. J. LeBlanc: Reproductive tract inflammatory disease in postpartum dairy cows. *Animal*, 8 Suppl 1, 54-63 (2014) doi:10.1017/S1751731114000524

55. R. S. Bisinotto, L. F. Greco, E. S. Ribeiro, N. Martinez, F. S. Lima, C. R. Staples, W. W. Thatcher and J. E. P. Santos: Influences of nutrition and metabolism on fertility of dairy cows. *Animal Reproduction*, 9(3), 260-272 (2012)

56. S. J. LeBlanc: Managing transition period health for reproductive performance in dairy cows. *Cattle Practice*, 21(3), 209-215 (2013)

57. A. Abuelo, J. Hernandez, J. L. Benedito and C. Castillo: A comparative study of the metabolic profile, insulin sensitivity and inflammatory response between organically and conventionally managed dairy cattle during the periparturient period. *Animal*, 8(9), 1516-1525 (2014)

58. L. Badinga, R. J. Collier, W. W. Thatcher and C. J. Wilcox: Effects of climatic and management factors on conception rate of dairy cattle in subtropical environment. *Journal of Dairy Science*, 68(1), 78-85 (1985) doi:10.3168/jds.S0022-0302(85)80800-6

59. J. K. Hillers, P. L. Senger, R. L. Darlington and W. N. Fleming: Effects of production, season, age of cow, days dry, and days in milk on conception to first service in large commercial. *Journal of Dairy Science*, 67(4), 861-867 (1985)

60. J. Zhang, L. X. Deng, H. L. Zhang, G. H. Hua and L. Han: Effects of parity on uterine involution and resumption of ovarian activities in postpartum Chinese Holstein dairy cows. *Journal of Dairy Science*, 95(5), 1979-86 (2010)

61. I. Prunner, H. Pothmann, K. Wagener, M. Giuliodori, J. Huber, M. Ehling-Schulz and M. Drillich: Dynamics of bacteriologic and cytologic changes in the uterus of postpartum dairy cows. *Theriogenology*, 82(9), 1316-22 (2014a) doi:10.1016/j.theriogenology.2014.08.018

62. O. B. Pascottini, M. Hostens, P. Sys, P. Vercauteren and G. Opsomer: Risk factors associated with cytological endometritis diagnosed at artificial insemination in dairy cows. *Theriogenology*, 92, 1-5 (2017b) doi:10.1016/j.theriogenology.2017.01.004

63. C. Inchaisri, H. Hogeveen, P. L. Vos, G. C. van der Weijden and R. Jorritsma: Effect of milk yield characteristics, breed, and parity on success of the first insemination in Dutch dairy cows. *Journal of Dairy Science*, 93(11), 5179-87 (2010b) doi:10.3168/jds.2010-3234

64. E. Collis, M. R. Fortes, Y. Zhang, B. Tier, K. Schutt, W. Barendse and R. Hawken: Genetic variants affecting meat and milk production traits appear to have effects on reproduction traits in cattle. *Animal Genetics*, 43(4), 442-6 (2012) doi:10.1111/j.1365-2052.2011.02272.x

65. S. Sasaki, Y. Uemoto, N. Sasago, T. Abe, S. Nishimura and Y. Sugimoto: Polymorphisms associated with four reproductive traits have no adverse effects on meat traits in Japanese Black cattle. *Animal Genetics*, 47(3), 386-7 (2016) doi:10.1111/age.12414

66. D. Sasaki, T. Ibi, T. Watanabe, T. Matsuhashi, S. Ikeda and Y. Sugimoto: Variants in the 3' UTR of General Transcription Factor IIF, polypeptide 2 affect female calving efficiency in Japanese Black cattle. *BMC Genetics*, 14, 41 (2013)

67. M. V. G. B. Silva, J. A. Cobuci, W. J. Ferreira, P. R. P. Oliveira, M. A. Machado and C. P. Ferreira: Effects of genetics and environment on the Mantiqueira type breed. I. Reproductive traits. . *Ciencia e Agrotecnologia*, 30(3), 522-528 (2006)

68. H. L. M. Moreira and R. A. Cardellino: Heritability, repeatability and environment effects in Hereford cattle. II. Calving interval, producing ability and cow productivity index. *Pesquisa Agropecuaria Brasileira*, 29(11), 1801-1805 (1994)

69. M. Penasa, N. López-Villalobos, R. D. Evans, A. R. Cromie, R. Dal Zotto and M. Cassandro: Crossbreeding effects on milk yield traits and calving interval in spring-calving dairy cows. *Journal of Animal Breeding and Genetics*, 127(4), 300-7 (2010) doi:10.1111/j.1439-0388.2009.00840.x

70. B. Faria e Vasconcellos, J. T. Padua, M. F. C. Munoz and H. Tonhati: Genetics and environmental effects on milk yield, calving interval and lactation length in a dairy crossbred herd. *Revista Universidade Rural*, 23(1), 39-45 (2003)

71. I. Prunner, K. Wagener, H. Pothmann, M. Ehling-Schulz and M. Drillich: Risk factors for uterine diseases on small- and medium-sized dairy farms determined by clinical, bacteriological, and cytological examinations. *Theriogenology*, 82(6), 857-65 (2014b) doi:10.1016/j.theriogenology.2014.06.015

72. O. Markusfeld: Factors responsible for post parturient metritis in dairy cattle. *Veterinary Record*, 114(22), 539-542 (1984)

73. M. T. Correa, H. Erb and J. Scarlett: Path analysis for seven postpartum disorders of Holstein cows. *Journal of Dairy Science*, 76(5), 1305-12 (1993) doi:10.3168/jds.S0022-0302(93)77461-5

74. I. M. Sheldon, S. B. Price, J. Cronin, R. O. Gilbert and J. E. Gadsby: Mechanisms of infertility associated with clinical and subclinical endometritis in high producing dairy cattle. *Reproduction in Domestic Animals*, 44 Suppl 3, 1-9 (2009b) doi:10.1111/j.1439-0531.2009.01465.x

75. F. D. Rensis and R. J. Scaramuzzi: Heat stress and seasonal effects on reproduction in the dairy cow—a review. *Theriogenology*, 60(6), 1139-1151 (2003) doi:10.1016/s0093-691x(03)00126-2

76. P. J. Hansen: Managing reproduction during heat stress in dairy cows. In: *Dairy production medicine*. Ed C. Risco&P. Melendez. John Wiley & Sons, (2011)

77. S. T. Butler: Nutritional management to optimize fertility of dairy cows in pasture-based systems. *Animal*, 8(1), 15-26 (2014) doi:10.1017/S1751731114000834

78. C. T. Westwood, I. J. Lean and J. K. Garvin: Factors influencing fertility of Holstein dairy cows: A multivariate description. *Journal of Dairy Science*, 85(12), 3225-3237 (2002) doi:DOI 10.3168/jds.S0022-0302(02)74411-1

79. F. Badinand: Les métrites chez la vache: influence des facteurs hormonaux et nutritionnels. *Cahiers de Medecine Veterinaire*, 44(5), 205-221 (1975)

80. S. Sato, T. Suzuki and K. Okada: Suppression of lymphocyte blastogenesis in cows with puerperal metritis and mastitis. *The Journal of Veterinary Medical Science*, 57(2), 373 (1995)

81. N. R. C. NRC: Nutrient requirements of dairy cattle. National Academic Press, Washington DC, USA (2001)

82. D. MacKay and A. L. Miller: Nutritional support for wound healing. *Alternative Medicine Review*, 8(4), 359-377 (2003)

83. E. Mayer: Relations entre alimentation et infécondité. *Le Point Vétérinaire* 78, 132 (1978)

84. H. M. Rutigliano, F. S. Lima, R. L. Cerri, L. F. Greco, J. M. Vilela, V. Magalhaes, F. T. Silvestre, W. W. Thatcher and J. E. Santos: Effects of method of presynchronization and source of selenium on uterine health and reproduction in dairy cows. *Journal of Dairy Science*, 91(9), 3323-36 (2008) doi:10.3168/jds.2008-1005

85. R. W. Bozworth, E. R. Bonewitz, E. P. Call and G. Ward: Analysis of factors affecting calving intervals of dairy cows. *Journal of Dairy Science*, 55(3), 334-338 (1972)

86. J. Hultgren and C. Svensson: Calving interval in dairy cows in relation to heifer rearing conditions in southwest Sweden. *Reproduction in Domestic Animals*, 45(1), 136-41 (2010) doi:10.1111/j.1439-0531.2008.01273.x

87. L. Elliot, K. J. Mcmahon, H. T. Gier and G. B. Marion: Uterus of the cow after parturition: bacterial content. *American Journal of Veterinary Research*, 29(1), 77-81 (1968)

88. A. M. Hussain, R. C. W. Daniel and D. O’Boyle: Postpartum uterine flora following normal and abnormal puerperium in cows. *Theriogenology*, 34(2), 291-302 (1990)

89. V. Zilaitis, A. Banys, R. Maruska and V. Ziogas: Diagnostic aspect of endometritis in cow. *Veterinarija ir Zootechnika*, 27, 41-44 (2004)

90. J. F. T. Griffin, P. J. Hartigan and W. R. Nunn: Non-specific uterine infection and bovine fertility: I. Infection patterns and endometritis during the first seven weeks post-partum. *Theriogenology*, 1(3), 91-106 (1974)

91. I. M. Sheldon, G. S. Lewis, S. LeBlanc and R. O. Gilbert: Defining postpartum uterine disease in cattle. *Theriogenology*, 65(8), 1516-30 (2006) doi:10.1016/j.theriogenology.2005.08.021

92. R. O. Gilbert, S. T. Shin, C. L. Guard, H. N. Erb and M. Frajblat: Prevalence of endometritis and its effects on reproductive performance of dairy cows. *Theriogenology*, 64(9), 1879-88 (2005) doi:10.1016/j.theriogenology.2005.04.022

93. L. V. Madoz, M. J. Giuliodori, A. L. Migliorisi, M. Jaureguiberry and R. L. de la Sota: Endometrial cytology, biopsy, and bacteriology for the diagnosis of subclinical endometritis in grazing dairy cows. *Journal of Dairy Science*, 97(1), 195-201 (2014) doi:10.3168/jds.2013-6836

94. S. J. LeBlanc, T. F. Duffield, K. E. Leslie, K. G. Bateman, G. P. Keefe, J. S. Walton and W. H. Johnson: Defining and diagnosing postpartum clinical endometritis and its impact on reproductive performance in dairy cows. *Journal of Dairy Science*, 85(9), 2223-2236 (2002)

95. P. C. Kennedy and R. B. Miller: Chapter 4: The female genital system. In. In: *Pathology of domestic animals*. Academic Press Limited, London, UK (1993)

96. S. Taylor: A review of equine sepsis. *Equine Veterinary Education*, 27(2), 99-109 (2015) doi:10.1111/eve.12290

97. G. N. Purohit, S. Ruhil and V. Khichar: Postpartum endometritis in dairy cows: current status of diagnosis, therapy and prevention. *Theriogenology Insight*, 5(1), 1-23 (2015) doi:10.5958/2277-3371.2015.00001.7

98. J. Dubuc, T. F. Duffield, K. E. Leslie, J. S. Walton and S. J. Leblanc: Definitions and diagnosis of postpartum endometritis in dairy cows. *Journal of Dairy Science*, 93(11), 5225-5233 (2010b) doi:0.3168/jds.2010-3428

99. S. McDougall, R. Macaulay and C. Compton: Association between endometritis diagnosis using a novel intravaginal device and reproductive performance in dairy cattle. *Animal Reproduction Science*, 99(1-2), 9-23 (2007) doi:10.1016/j.anireprosci.2006.03.017

100. L. V. Madoz, R. L. De la Sota, K. Suzuki, W. Heuwieser and M. Drillich: Use of hysteroscopy for the diagnosis of postpartum clinical endometritis in dairy cows. *Veterinary Record*, 167(4), 142-3 (2010) doi:10.1136/vr.c3157

101. S. Pleticha, M. Drillich and W. Heuwieser: Evaluation of the Metricheck device and the gloved hand for the diagnosis of clinical endometritis in dairy cows. *Journal of Dairy Science*, 92(11), 5429-35 (2009) doi:10.3168/jds.2009-2117

102. M. A. Rahman, M. G. S. Alam and M. Shamsuddin: Histopathological evaluation of the uterine biopsies diagnostic to different level of clinical endometritis in dairy cows. *Bangladesh Veterinarian*, 19(2), 77-85 (2002)

103. R. Fournier, L. Deguillaume and S. Chastant-Maillard: What is the preferred method in practice for the diagnosis of endometritis in dairy cows? *Point Veterinaire*, 45(349), 66-71 (2014)

104. G. S. Lewis: Uterine health and disorders. *Journal of Dairy Science*, 80(5), 984-994 (1997) doi:10.3168/jds.S0022-0302(97)76024-7

105. K. Bretzlaff: Rationale for treatment of endometritis in the dairy cow. *Veterinary Clinics of North America*, 3(3), 593-607 (1987)

106. A. T. Peter, G. M. Jarratt and D. W. Hanlon: Accuracy of diagnosis of clinical endometritis with Metricheck^TM^ in postpartum dairy cows. *Clinical Theriogenology*, 3(4), 461-465 (2011)

107. S. Westermann, M. Drillich, T. B. Kaufmann, L. V. Madoz and W. Heuwieser: A clinical approach to determine false positive findings of clinical endometritis by vaginoscopy by the use of uterine bacteriology and cytology in dairy cows. *Theriogenology*, 74(7), 1248-55 (2010) doi:10.1016/j.theriogenology.2010.05.028

108. C. Leutert, X. von Krueger, J. Plöntzke and W. Heuwieser: Evaluation of vaginoscopy for the diagnosis of clinical endometritis in dairy cows. *Journal of Dairy Science*, 95(1), 206-12 (2012) doi:10.3168/jds.2011-4603

109. D. J. Runciman, G. A. Anderson, J. Malmo and G. M. Davis: Use of postpartum vaginoscopic (visual vaginal) examination of dairy cows for the diagnosis of endometritis and the association of endrometritis with reduced reproductive performance. *Australian Veterinary Journal*, 86(6), 205-13 (2008) doi:10.1111/j.1751-0813.2008.00301.x

110. H. Okawa, A. Fujikura, M. M. P. Wijayagunawardane, P. L. A. M. Vos, M. Taniguchi and M. Takagi: Effect of diagnosis and treatment of clinical endometritis based on vaginal discharge score grading system in postpartum holstein cows. *Journal of Veterinary Medical Science*, 79(9), 1545-1551 (2017)

111. I. M. Sheldon, D. E. Noakes, A. N. Rycroft and H. Dobson: Effect of postpartum manual examination of the vagina on uterine bacterial contamination in cows. *Veterinary Record*, 151(18), 531-534 (2002)

112. M. L. Bicalho, F. S. Lima, V. S. Machado, E. B. Meira, Jr., E. K. Ganda, C. Foditsch, R. C. Bicalho and R. O. Gilbert: Associations among Trueperella pyogenes, endometritis diagnosis, and pregnancy outcomes in dairy cows. *Theriogenology*, 85(2), 267-74 (2016) doi:10.1016/j.theriogenology.2015.09.043

113. S. Pedersen, M. Sheldon, M. Burnell, R. Smith and M. Kerby: Uterine disease in dairy cattle: monitoring and management. *Livestock*, 18(5), 150-157 (2013)

114. E. S. Ribeiro, F. S. Lima, L. F. Greco, R. S. Bisinotto, A. P. Monteiro, M. Favoreto, H. Ayres, R. S. Marsola, N. Martinez, W. W. Thatcher and J. E. Santos: Prevalence of periparturient diseases and effects on fertility of seasonally calving grazing dairy cows supplemented with concentrates. *Journal of Dairy Science*, 96(9), 5682-97 (2013) doi:10.3168/jds.2012-6335

115. W. Barański, M. Podhalicz-Dzięgielewska, S. Zduńczyk and T. Janowski: The diagnosis and prevalence of subclinical endometritis in cows evaluated by different cytologic thresholds. *Theriogenology*, 78(9), 1939-47 (2012) doi:10.1016/j.theriogenology.2012.07.018

116. A. Dourey, M. G. Colazo, P. P. Barajas and D. J. Ambrose: Relationships between endometrial cytology and interval to first ovulation, and pregnancy in postpartum dairy cows in a single herd. *Research in Veterinary Science*, 91(3), e149-53 (2011) doi:10.1016/j.rvsc.2010.11.011

117. G. Opsomer, Y. Grohn, J. Hertl, M. Coryn, H. Deluyker and A. de Kruif: Risk factors for post partum ovarian dysfunction in high producing dairy cows in Belgium: A field study. *Theriogenology*, 53(4), 841-857 (2000)

118. M. Gobikrushanth, R. Salehi, D. J. Ambrose and M. G. Colazo: Categorization of endometritis and its association with ovarian follicular growth and ovulation, reproductive performance, dry matter intake, and milk yield in dairy cattle. *Theriogenology*, 86(7), 1842-9 (2016) doi:10.1016/j.theriogenology.2016.06.003

119. J. Denis-Robichaud and J. Dubuc: Determination of optimal diagnostic criteria for purulent vaginal discharge and cytological endometritis in dairy cows. *Journal of Dairy Science*, 98(10), 6848-55 (2015) doi:10.3168/jds.2014-9120

120. O. B. Pascottini, M. Hostens, P. Sys, P. Vercauteren and G. Opsomer: Cytological endometritis at artificial insemination in dairy cows: Prevalence and effect on pregnancy outcome. *Journal of Dairy Science*, 100(1), 588-597 (2017b) doi:10.3168/jds.2016-11529

121. T. Janowski, S. Zduńczyk and W. Barański: Subclinical endometritis as a cause of insemination failure in dairy cows. *Medycyna Weterynaryjna*, 67(2), 79-82 (2011)
