# Supplementary material for: The Effect of Abnormal Reproductive Tract Discharge on the Calving to Conception Interval of Dairy Cows
Source: Front Vet Sci. 2019 Oct 22;6:374. doi: 10.3389/fvets.2019.00374 (PMC6817506; doi:10.3389/fvets.2019.00374)
Supplement: Supplementary Dataset 2 — log-cumulative hazard plots and observed vs. predicted hazard plots of cows with and without abnormal reproductive tract discharge (ARTD), and plots of Schoenfeld residuals of variables in Cox proportional hazards models to determine the association between ARTD and the calving to conception interval of cows in a study to identify the influence of ARTD and other potential risk factors on the calving to conception interval in dairy cows on three farms in the Riverina, NSW, Australia. [file Data_Sheet_2.docx]

Supplementary Datasheet 2


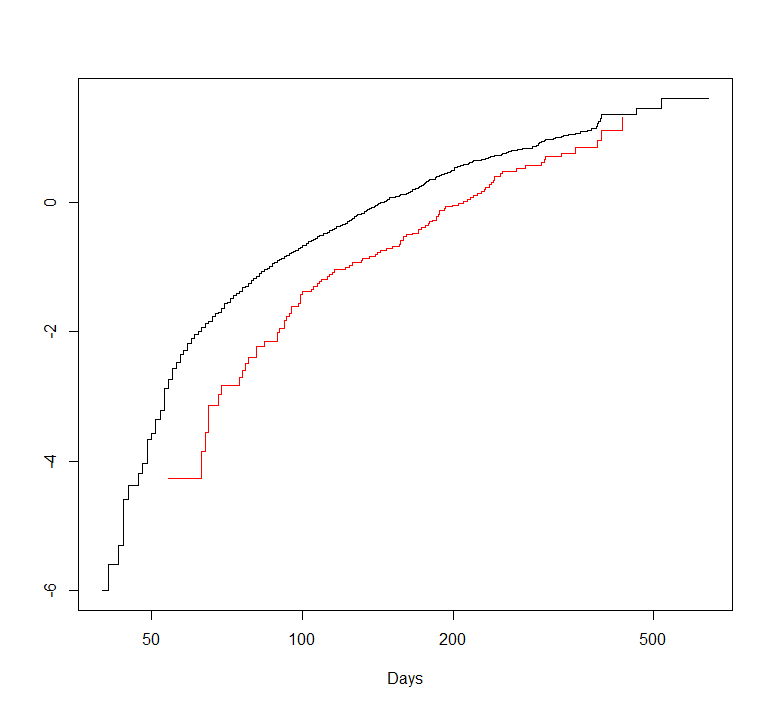


Figure A: Log cumulative hazard plot of cows with (red line) and without endometritis in a study to identify the influence of endometritis and other potential risk factors on the calving to conception interval in dairy cows on three farms in the Riverina, NSW, Australia.


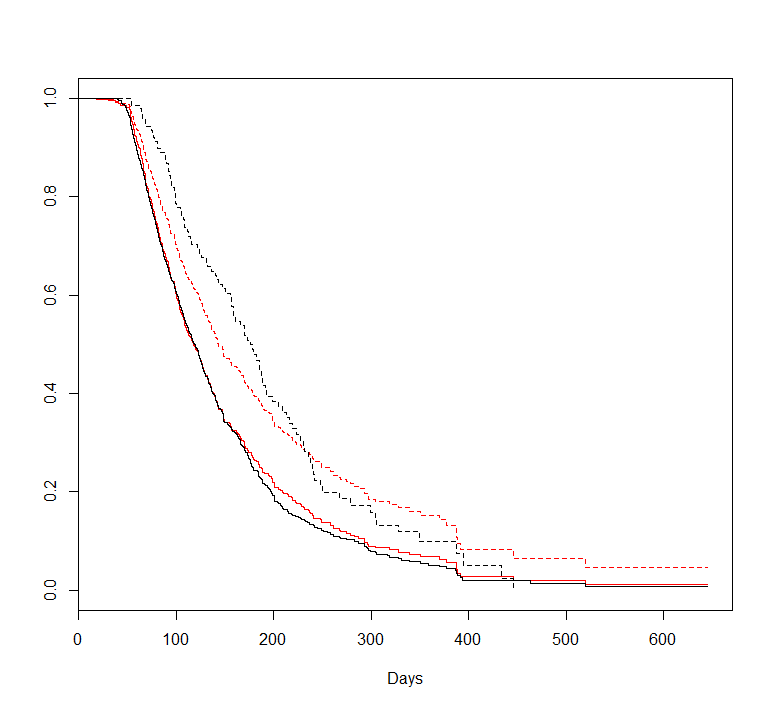


Figure B: Observed (black) and predicted (red) Kaplan-Meier survival curves of the probability of remaining not in-calf (y axis), dependent on the presence of abnormal reproductive tract discharge (ARTD; dotted line = ARTD positive) during lactation in a study to identify the influence of ARTD and other potential risk factors on the calving to conception interval in dairy cows on three farms in the Riverina, NSW, Australia.


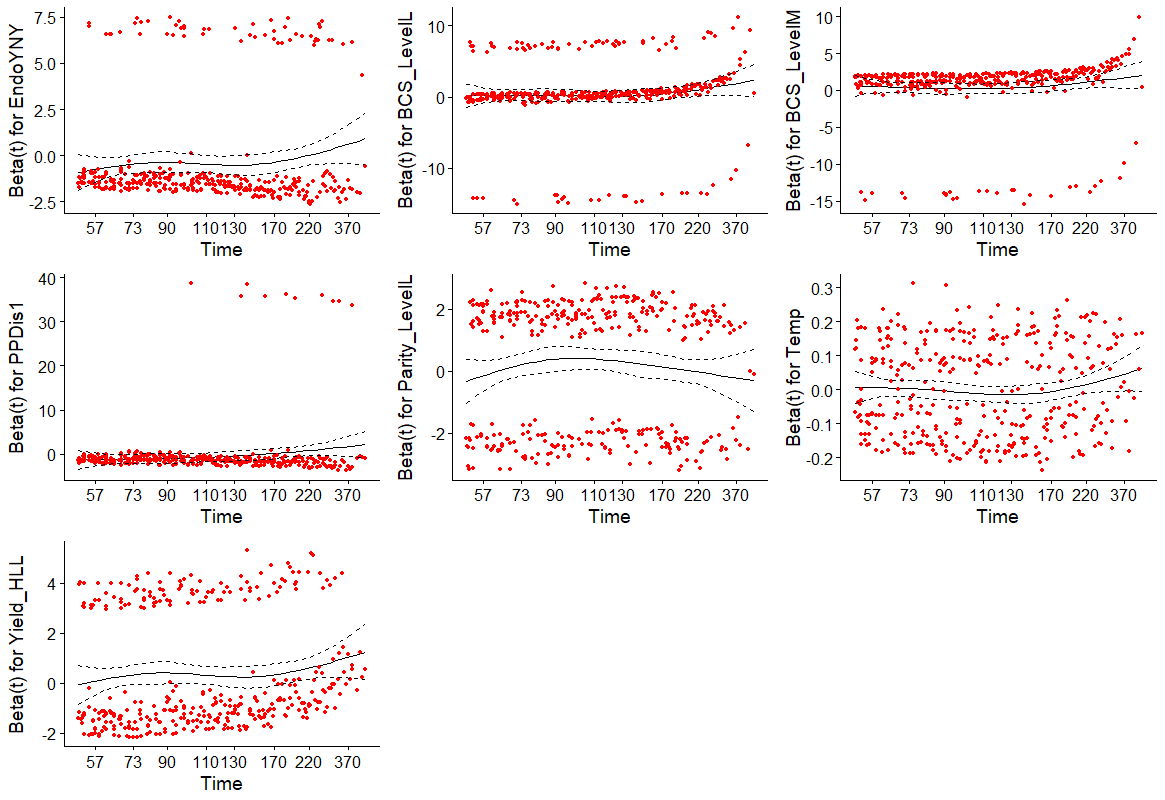


Figure C: Schoenfeld residuals from Cox proportional hazards model of calving to conception interval (outcome) and the direct effects of endometritis (predictor; EndoYNY), with covariates body condition score (BCS), peri-parturient disease (PPDis), parity, environmental temperature and milk yield (Yield) in a study to identify the influence of ARTD and other potential risk factors on the calving to conception interval in dairy cows on three farms in the Riverina, NSW, Australia.


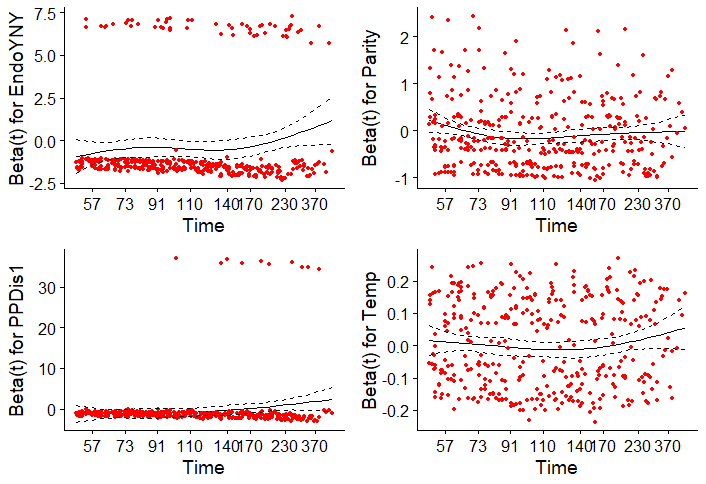


Figure D: Schoenfeld residuals from Cox proportional hazards model of calving to conception interval (outcome) and the total effects of endometritis (predictor; EndoYNY), with covariates parity, peri-parturient disease (PPDis), environmental temperature and milk yield (Yield) in a study to identify the influence of ARTD and other potential risk factors on the calving to conception interval in dairy cows on three farms in the Riverina, NSW, Australia.
