# Supplementary figures and images for: The Effect of Abnormal Reproductive Tract Discharge on the Calving to Conception Interval of Dairy Cows
Source: Front Vet Sci. 2019 Oct 22;6:374. doi: 10.3389/fvets.2019.00374 (PMC6817506; doi:10.3389/fvets.2019.00374)

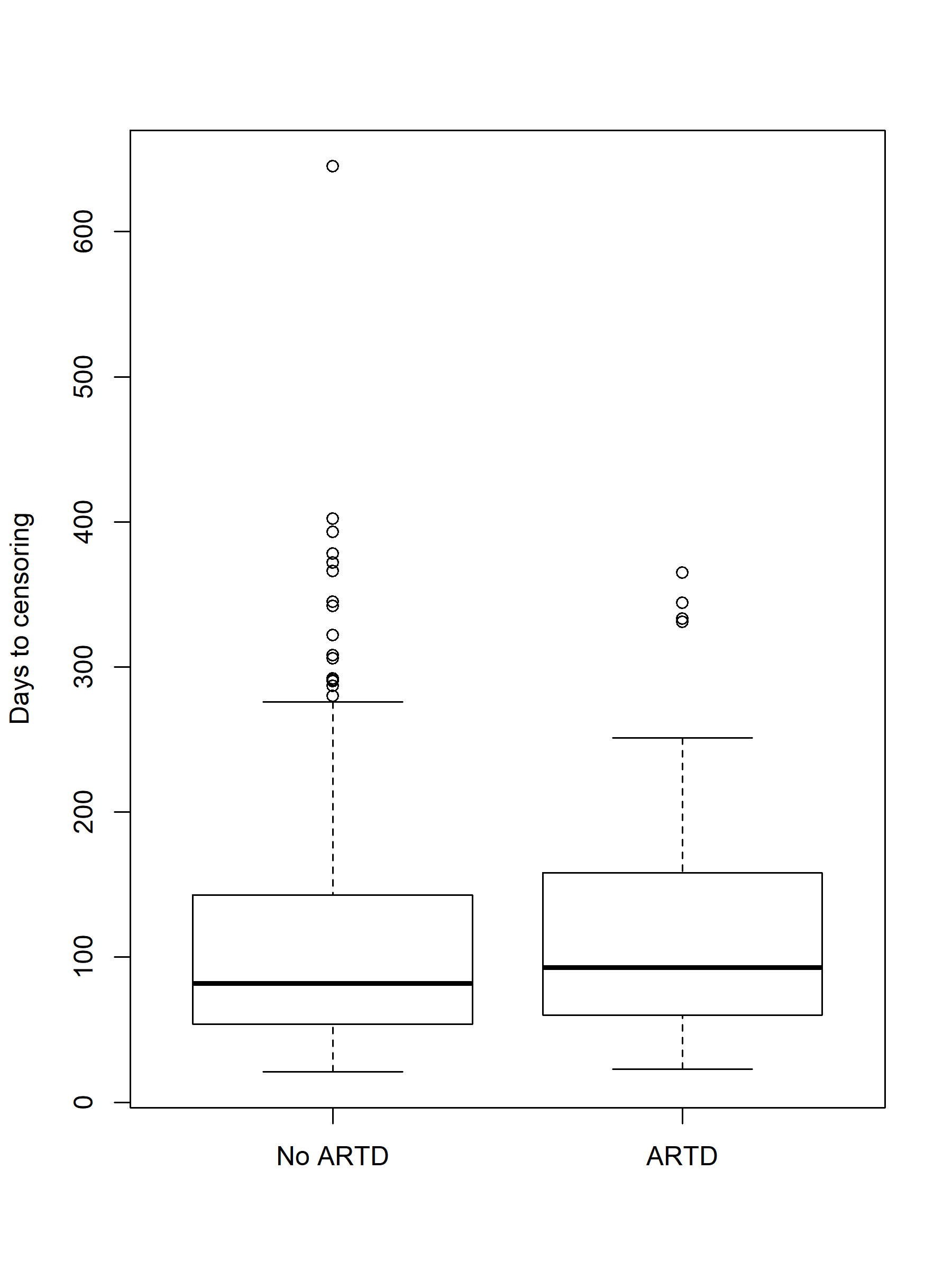

Supplement: Supplementary Figure 1 — Boxplots of time to right censoring in cows with and without abnormal reproductive tract discharge (ARTD) in a study to identify the influence of ARTD and other potential risk factors on the calving to conception interval in a study of dairy cows on three farms in the Riverina, New South Wales, Australia. [file Image_1.TIFF]

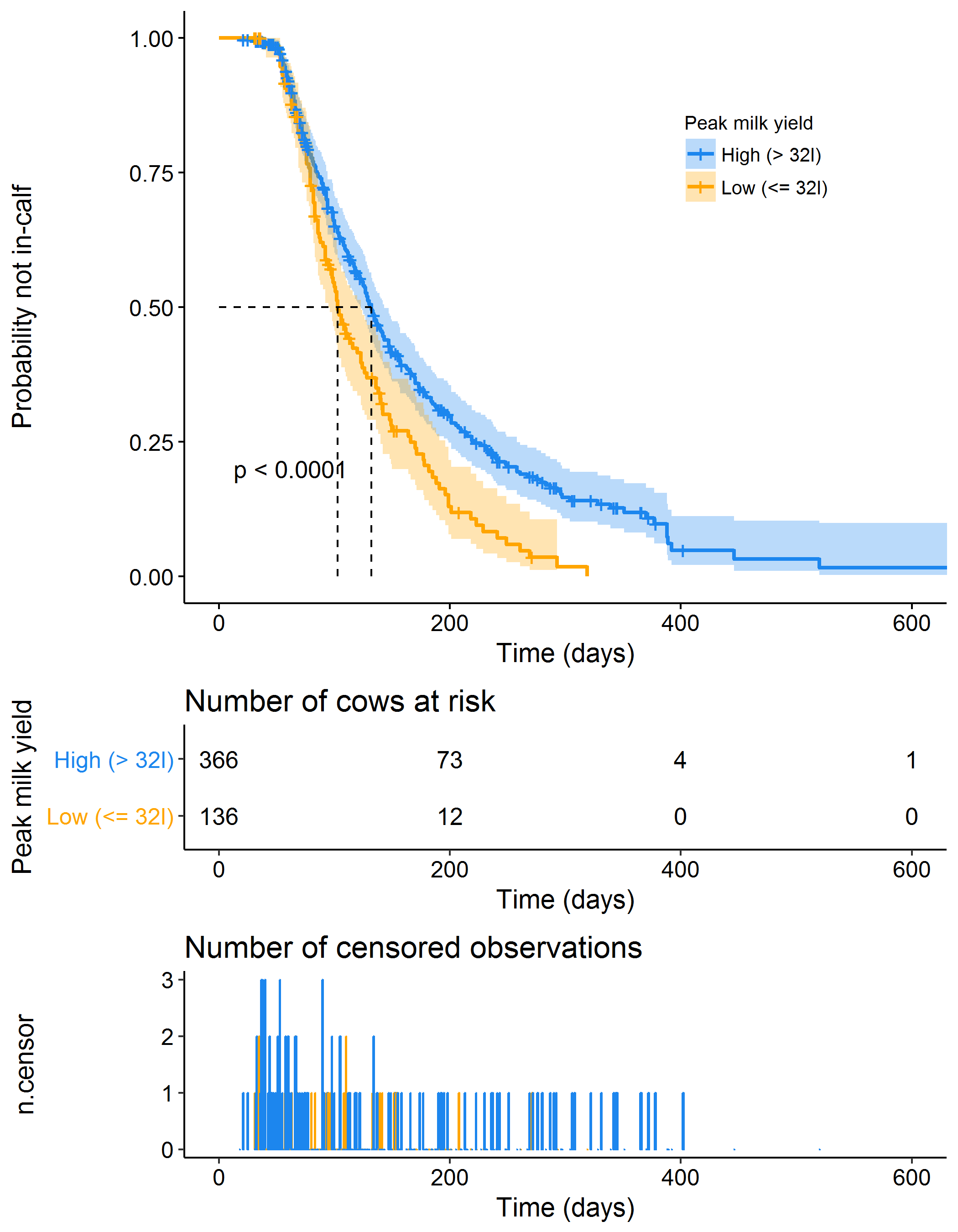

Supplement: Supplementary Figure 2 — Kaplan-Meier survival curve of the probability of remaining not in-calf dependent on peak milk yield. The table of “Number of cows at risk” shows the number of cows that remained non-pregnant at time points following calving. The graph of the “Number of censored cows” shows the number of cows that did not conceive or were not detected as having conceived in the allocated study period (orange = cows with low peak milk yield). [file Image_2.TIFF]

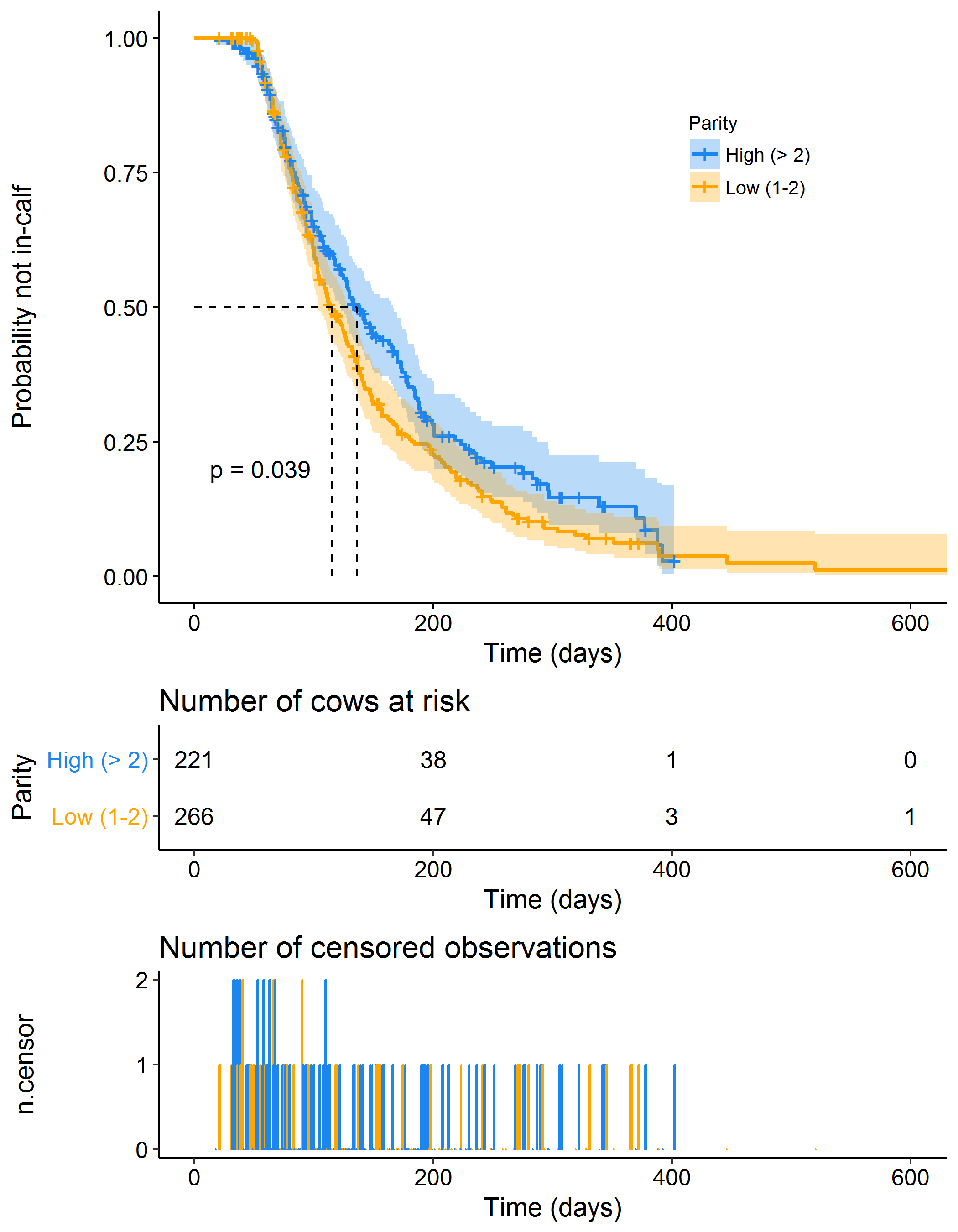

Supplement: Supplementary Figure 3 — Kaplan-Meier survival curve of the probability of remaining not in-calf dependent on parity. The table of “Number of cows at risk” shows the number of cows that remained non-pregnant at time points following calving. The graph of the “Number of censored cows” shows the number of cows that did not conceive or were not detected as having conceived in the allocated study period (orange = cows with low peak milk yield). [file Image_3.TIFF]
